# Unveiling the Bovine Epimural Microbiota Composition and Putative Function

**DOI:** 10.3390/microorganisms9020342

**Published:** 2021-02-09

**Authors:** Cátia Pacífico, Renée Maxine Petri, Sara Ricci, Elsayed Mickdam, Stefanie Urimare Wetzels, Viktoria Neubauer, Qendrim Zebeli

**Affiliations:** 1 Christian Doppler Laboratory for Innovative Gut Health Concepts of Livestock, Department for Farm Animals and Veterinary Public Health, Institute of Animal Nutrition and Functional Plant Compounds, University of Veterinary Medicine, 1210 Vienna, Austria; sara.ricci@vetmeduni.ac.at (S.R.); qendrim.zebeli@vetmeduni.ac.at (Q.Z.); 2 Sherbrooke Research and Development Centre, Agriculture and Agri-Food Canada, Sherbrooke, QC J1M1Z7, Canada; renee.petri@canada.ca; 3 Nutrition and Clinical Nutrition Department, Faculty of Veterinary Medicine, South Valley University, Qena 83523, Egypt; e.mickdam@vet.svu.edu.eg; 4 Unit for Food Microbiology, Department for Farm Animals and Veterinary Public Health, Institute of Food Safety, Food Technology and Veterinary Public Health, University of Veterinary Medicine, 1210 Vienna, Austria; stefanie.wetzels@vetmeduni.ac.at (S.U.W.); viktoria.neubauer@vetmeduni.ac.at (V.N.); 5 Austrian Competence Centre for Feed and Food Quality, Safety and Innovation FFoQSI GmbH, 3430 Tulln, Austria

**Keywords:** rumen papillae, epimural microbiota, bovine rumen epithelium, core microbiota

## Abstract

Numerous studies have used the 16S rRNA gene target in an attempt to characterize the structure and composition of the epimural microbiota in cattle. However, comparisons between studies are challenging, as the results show large variations associated with experimental protocols and bioinformatics methodologies. Here, we present a meta-analysis of the rumen epimural microbiota from 11 publicly available amplicon studies to assess key technical and biological sources of variation between experiments. Using the QIIME2 pipeline, 332 rumen epithelial microbiota samples were analyzed to investigate community structure, composition, and functional potential. Despite having a significant impact on microbial abundance, country of origin, farm, hypervariable region, primer set, animal variability, and biopsy location did not obscure the identification of a core microbiota. The bacterial genera *Campylobacter*, *Christensenellaceae* R-7 group, *Defluviitaleaceae* UCG-011, *Lachnospiraceae* UCG-010, *Ruminococcaceae* NK4A214 group, *Ruminococcaceae* UCG-010, *Ruminococcaceae* UCG-014, *Succiniclasticum*, *Desulfobulbus*, and *Comamonas* spp. were found in nearly all epithelium samples (>90%). Predictive analysis (PICRUSt) was used to assess the potential functions of the epithelial microbiota. Regularized canonical correlation analysis identified several pathways associated with the biosynthesis of precursor metabolites in *Campylobacter, Comamonas*, *Desulfobulbus*, and *Ruminococcaceae* NK4A214, highlighting key metabolic functions of these microbes within the epithelium.

## 1. Introduction

The rumen epimural bacteria refers to the microbiota that are firmly attached to the rumen wall [1,2]. Despite contributing to less than 1% of the overall rumen bacterial biomass [3], these bacteria play a critical role in host physiological development, tissue turnover, urea recycling, and competitive exclusion of transient pathogens [3,4,5,6,7,8]. Within the epimural population, microbes also confer protection to the epithelium from challenging ruminal conditions such as low pH, osmotic stress, and pathogen-blooms associated with ruminal dysbiosis [9,10]. However, unlike the feed and fluid-associated rumen microbiota, little is known about the impact of the host, age, breed, gender, and geographic location on the rumen epimural community, or whether there is a definitive core microbiota associated with the rumen epithelium. The current definition is that a core microbiota is not found at any particular taxonomic level, but instead is the core set of genes available to a community for performing necessary metabolic functions associated with its niche [11,12]. This is critical information for being able to define a healthy ecosystem, as well as to understand dysbiosis. Since modern feeding practices in cattle often create digestive disturbances, the stability and the metabolic functions of those microbiota at the interface between the digestive milieu and the host tissue are of great interest. Specifically, the rumen epithelium is densely populated with *Proteobacteria*, most of them phylogenetic neighbors to pathogenic species [13]. It is therefore plausible that a dysbiotic epithelial community would have the greatest potential for increased numbers of opportunistic pathogens and their derived products with high pro-inflammatory properties (i.e., endotoxins) that induce local and systemic inflammation and other health disturbances [14]. Consequently, it is necessary to define a core epithelial microbiota and describe its diversity and function to better understand and assess dysbiosis.

Compounding this lack of knowledge is a lack of standardization in the non-culture-based methods of analysis. The rapid advancement of the fields of sequencing and bioinformatics has created a large disparity in the methods of analysis, making it difficult to interpret data and advance the field of rumen microbiology. Technical differences are mainly associated with the selection of the amplified 16S rRNA hypervariable region, the use of different sets of PCR primers, DNA extraction protocols, sequencing platforms, and bioinformatics workflows [12,15,16]. It is essential that methodologies are standardized, and bioinformatics are closely assessed to ensure that accurate and comparable data is presented for interpretation and analysis. Therefore, a meta-analysis of all publicly available datasets encompassing the rumen bovine epimural community was performed to assess the weight that various environmental and analytical factors have on the data obtained from 16S rRNA sequencing.

## 2. Materials and Methods

### 2.1. Search Overview, Inclusion Criteria and Data Acquisition

All available studies related to cow epimural microbiota were systematically reviewed. The literature search included all studies in NCBI PubMed (http://www.ncbi.nlm.nih.gov/pubmed (accessed on 30 April 2020)) and Google Scholar (http://scholar.google.com (accessed on 30 April 2020)) that included (i) the terms “papillae”, “epithelium, OR “epimural”, (ii) published since 1998, (iii) in “cow” OR “bovine” OR “cattle” (iv) and using “amplicon” OR “16S rRNA” sequencing data. Additionally, NCBI BioProject (http://www.ncbi.nlm.nih.gov/bioproject (accessed on 30 April 2020)) was also screened for unpublished data. Sequence files from each study were downloaded from SRA (http://www.ncbi.nlm.nih.gov/sra (accessed on 30 April 2020)) or obtained directly from authors of previous studies. Our final dataset contained 16S rRNA gene sequences of rumen wall samples in cattle from 11 previously published datasets and 332 samples (Table 1). The datasets were further merged into 8 projects when belonging to the same study.

### 2.2. Datasets

Studies belonging to the same sequencing project were grouped together. These included projects conducted in Austria [9,10,17,18,19,20], the USA [21,22], Ireland (unpublished; PRJNA484585), Belgium [23], and Germany [24]. Studies conducted in Austria [9,10], [17,18,19,20] were all conducted at the same farm and in some cases using the same animals. Two studies conducted in the USA were conducted in different farms [21,22]. All projects included purebred Holstein-Friesian dairy cows, with the exception of the study from Ireland which used Holstein and Charolais steers (unpublished; PRJNA484585). Biopsy samples were collected from several locations: ventral sac [9,10,17,18,19,20,21,23,24], (unpublished; PRJNA484585), dorsal sac [22], cranial sac [21], caudoventral blind sac [21], and caudodorsal blind sac [21]. All studies used primers sets for Illumina paired-end sequencing of the V4 [21,22,24], V3–4 [23], and V3–5 [9,10,17,18,19,20] hypervariable regions (Table 2). Different DNA extraction methods were used, either based on kits [9,10], [17,18,19,20,22,24] or phenol-chloroform extraction methods [21,23], with [9,10,17,18,19,20,21,22,23] or without bead beating [24]. None of the studies reported the inclusion of positive controls e.g., a mock community or an extraction/reagent (negative) control.

### 2.3. Sequence Analysis

Raw sequences from each project were analyzed and checked for sequence quality and adapter contamination using FASTQC [25] and AdapterRemoval [26]. Microbial community analysis was performed with QIIME2 v2020.2 [27]. Paired-end sequence data was merged using vsearch join-pairs [28] with the option —p-maxee 2 and quality filtered using the q-score-joined plugin with a minimum acceptable PHRED score of 20 (—p-min-quality 20). For some datasets (ADDA and Sugarhay), these criteria had to be adjusted in order to allow more input reads (Supplementary Table S1). Denoising was performed with deblur [29] using a—p-trim-length of 200 bp. Representative sequences and feature tables obtained for each project were merged and filtered in order to exclude all sub-operational taxonomic units (sOTU) classified as mitochondria or chloroplast sequences. Taxonomy was assigned to sOTU using a classify-sklearn naive Bayes taxonomy classifier against the SILVA 132 99% OTUs reference sequences [30], resulting in a total of 56,025 features. Alpha- and beta-diversity metrics were estimated after samples were rarefied to 8000 sequences per sample to account for uneven sequencing depth, resulting in the exclusion of 124 samples and two entire studies [23,24]. Core microbiota was determined by extracting the core features at phylum, family, genus, and sOTU level in at least 90% of the samples using the compute_core_microbiome.py command in QIIME 1.9.1. [31]. The final sOTU table was used for functional metagenomic prediction using PICRUSt2 [32]. The pathway level predictions are given based on MetaCyc [33] pathways and IDs (Supplementary Table S2). To determine pairwise associations between core microbiota and predicted functions, regularized canonical correlation analysis was performed using the R package “mixOmics” (version 6.12.2) in R studio (version 4.0.2) [34].

### 2.4. Statistical Evaluation of Data

Alpha diversity metrics were checked for normality using the Shapiro-Wilk test (*p* < 0.05) and compared among studies using a non-parametric Kruskal-Wallis test with a pairwise Wilcoxon rank sum test in the R software environment [34]. Significance was considered at a *p*-value of < 0.05 after correction with the Benjamin-Hochberg procedure [35]. Principal-coordinate analysis (PCoA) was used to visualize distances between beta-diversity matrices. Permutational multivariate analysis of variance (PERMANOVA) using adonis [36] with 999 permutations and analysis of similarities (ANOSIM) was used to analyze the unweighted and weighted UniFrac distances and the Bray-Curtis dissimilarities matrices regarding study, country of origin, farm, gender (cow vs. steer), individual variability, biopsy location, hypervariable region, and primer set used. Permutational analysis of multivariate dispersions (PERMDISP) was used to test the homogeneity of dispersion for each metadata category analyzed previously [36]. Linear discriminant analysis effect size (LEfSe) (https://huttenhower.sph.harvard.edu/galaxy/ (accessed on 30 April 2020), The Huttenhower Lab, Boston, MA, USA) was used to determine which genera were significantly enriched according to the hypervariable region sequenced, and which metabolic pathways were overestimated in the different rumen locations. Genera and pathways that were more abundant in a particular group were identified by LEfSe using the non-parametric factorial Kruskal-Wallis (KW) sum-rank test (*p* < 0.05) and the effect size of each of these genera was estimated using linear discriminant analysis [37]. A LDA score (log10) of 4.0 was used as the cut-off to identify differentially abundant microbial genera and a score of 3.0 was used to identify differentially abundant metabolic pathways in the different rumen locations.

## 3. Results and Discussion

### 3.1. Composition and Structure of the Core Epimural Microbiota

**Figure 1:**
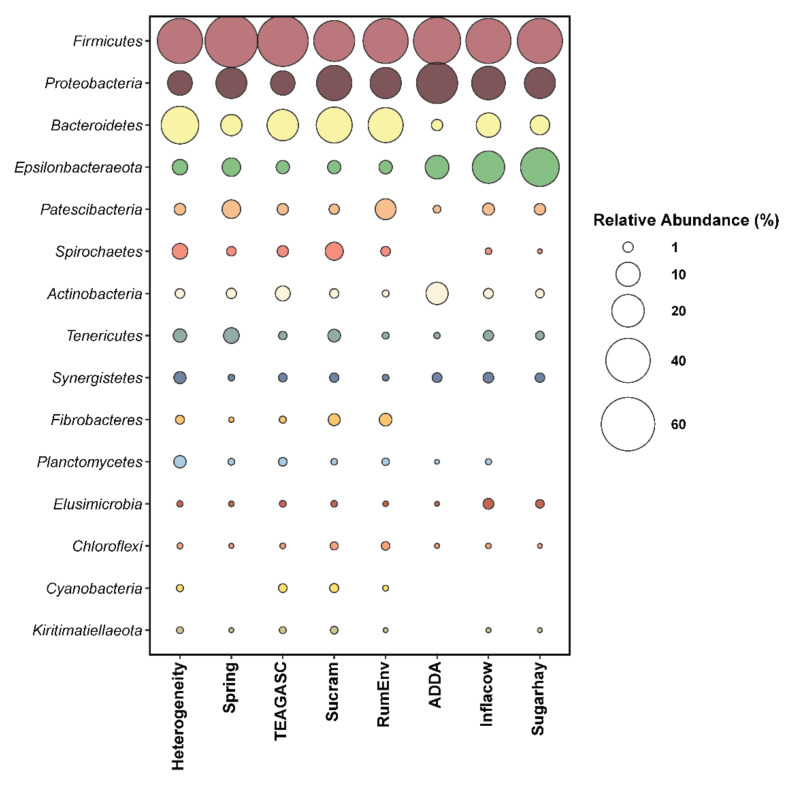
Percentage of most abundant phyla (relative abundance > 0.1%) found in each study.

**Figure 2:**
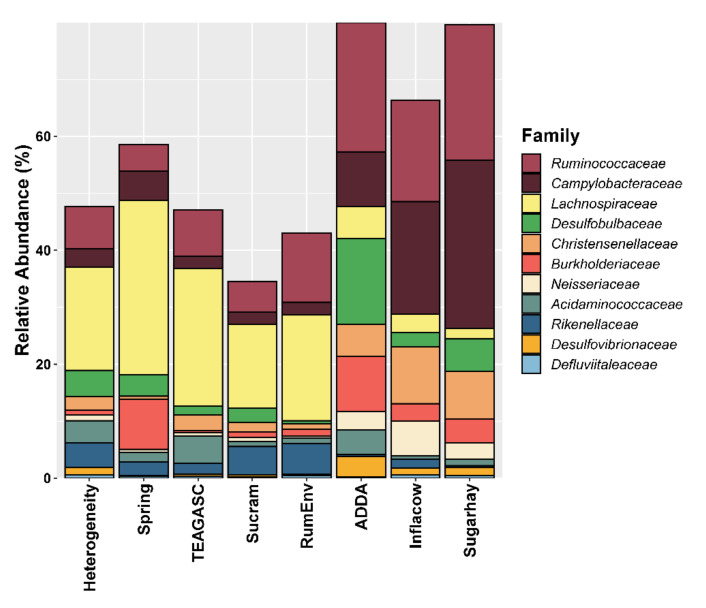
Relative abundance of core bacterial families found in > 90% of the samples per study.

We were able to identify 11 studies, grouped in 8 datasets, that met the search criteria used (Table 1 and Table 2) comprising of a total of 332 samples. Core microbiota was defined as the taxa present in 90% of the samples, allowing the identification of 6 phyla (Figure 1), 11 families (Figure 2), and 11 genera as fundamental representatives of the epithelial microbiota.

### 3.2. Core Bacterial Phyla Are Shared across Studies

Only *Firmicutes, Proteobacteria*, and *Epsilonbacteraeota* were present in each single sample analyzed. The latter is a newly proposed phyla, that derives from the combination of *Epsilonproteobacteria*, formerly included in *Proteobacteria*, and of the order *Desulfurellales (Deltaproteobacteria)* [38]. The same authors recently proposed to replace the name of this new phylum with *Campylobacterota* [38,39], given that *Campylobacter* is one of its most widely known members. The six core phyla detected correspond to the most abundant phyla across all the studies, with the exception of *Spirochaetes*, which was not identified in the project ADDA [18,19]. While Neubauer et al. [19] identified this phylum only in the particle-associated microbiota, Petri et al. [18] found *Spirochaetes* to be present at low abundance on the ruminal epithelium. Since the methods were extremely similar for both studies, the presence or absence of this phylum could be due to the bioinformatics analyses performed. *Proteobacteria* and *Firmicutes*, in different proportions, were the most abundant phyla across all the studies analyzed. In accordance with our findings, the studies from De Mulder and Neubauer [19,23] found *Bacteroidetes* to be less present in the epimural niche compared to other ruminal microenvironments.

### 3.3. Lower Taxonomical Levels Allow the Identification of a Core

**Figure 3:**
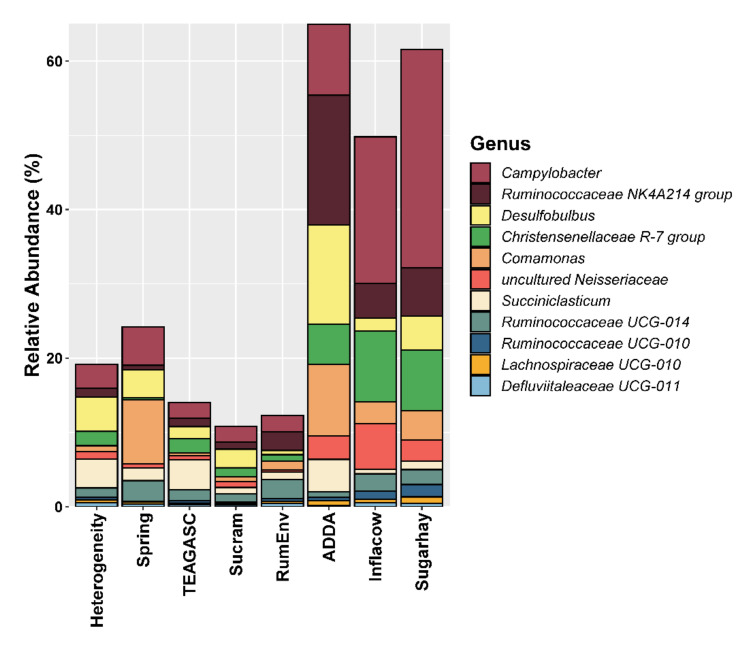
Relative abundance of core bacterial genera found in > 90% of the samples per study.

*Campylobacteraceae* was represented in 100% of the samples, confirming the importance of the recently proposed *Campylobacterota* phylum in the epimural fraction of the rumen microbiota. Interestingly, *Campylobacter* (12.1 ± 0.66%) was also the only genus to be detected in all the samples and was also the most commonly described genus across all studies included in this meta-analysis (Figure 3). Out of 344 bacterial genera, *Campylobacter* (15.5%), *Kingella* (7.8%), *Desulfobulbus* (4.7%), and *Brachymonas* (4.2%) were previously found to be the most abundant epimural bacteria [9]. Project Inflacow described the most abundant epithelial OTUs to be *Campylobacter hyointestinalis*, followed by *Kingella oralis* [9,10]. Results of our meta-analysis partially confirm these findings, as the feature classified as genus *Campylobacter* is 100% similar to *Campylobacter hyointestinalis* (NCBI Accession number: NR_115710.1). As previously discussed, most of the studied members of the *Campylobacteraceae* family are pathogenic strains, such as *C. jejuni* [40,41]. However, the exact role of these microbes in the epithelial community remains unknown. No other studies report the presence of *K. oralis*, which might suggest that this is a bioinformatics artifact or a study-specific effect. Two features identified as an uncultured *Neisseriaceae* (2.79 ± 0.19%) are classified in the NCBI database as *Neisseria oralis* (NCBI Accession number: NR_118249.1). *Neisseria* has been previously found to be associated with oxidative stress response in a transcriptome study of the cattle epimural microbiota using the same samples as Inflacow [42]. Likewise, most of the literature concerning *Neisseriaceae* refers to pathogenic bacteria. Members of this family are found in many different animal species, and a few strains are associated with microbiological communities populating the oral cavity in ruminants [43]. Some species, such as *N. sicca*, have been suggested to have urea hydrolysis activity in the rumen [44], whereas *N. oralis* has been demonstrated to be able to ferment lactose [45].

Features belonging to *Ruminococcaceae* UCG-014 (1.55 ± 0.07%), *Ruminococcaceae* UCG-010 (0.69 ± 0.04%), and *Ruminococcaceae* NK4A214 (6.76 ± 0.41%) group were identified as core in this meta-analysis, and have been previously reported as present in the whole gastrointestinal (GI) tract of cattle [46]. The latter is a relatively new genus, which has been recently added to the SILVA database, as it was previously included in broader classification at the family level [47]. Therefore, this group was not described in any of the studies included in this meta-analysis, as it was before considered among the *Ruminococcaceae*. Although bacteria belonging to this family are well known for their fibrolytic activity, *Ruminococcaceae* NK4A214 group were identified and cultured for the first time in 2011 [48], and their exact function in the rumen is still unknown. *Burkholderiaceae* is a Gram-negative family with an incredible ecological and metabolic variability, with several species classified as pathogens for humans, animals, and plants. Their exact role in epimural ruminal microbial community requires further investigation, but their capacity to adapt to challenging conditions could explain their presence in the core epimural microbiota [49], making the genus *Comamonas* (4.53 ± 0.32%) a very interesting epimural microbe. Family *Desulfovibrionaceae*, also identified as core, being able to reduce sulfate and produce acetate through the metabolism of lactate and hydrogen [50,51]. Families *Lachnospiraceae* and *Desulfobulbaceae*, identified as highly prevalent in three studies, [21,23,24], were confirmed as part of the core microbiota in our study. *Lachnospiraceae* UCG-010 (0.46 ± 0.02%) was also identified as part of the core microbiota. *Lachnospiraceae* are Gram-positive bacteria responsible for pectin fermentation and formate production [52], according to some authors. In other studies, the known role as fibrolytic bacteria [12,53] of unclassified *Lachnospiraceae* together with unclassified *Ruminococcaceae* was confirmed by being positively correlated with acetate production and an increased acetate to propionate ratio [54]. Despite being frequently identified as part of the ruminal microbiota, family *Desulfobulbaceae* is rarely examined and discussed. Some of its components, in particular belonging to the genus *Desulfobulbulbus* (5.79 ± 0.35%), have been described as sulfur compounds reducing and oxygen scavenging bacteria [23,24].

In contrast with many other genera, the role of *Succiniclasticum* (2.50 ± 0.18%) in the rumen seems to be clear: bacteria belonging to this group convert succinate to propionate, which is the most important source for gluconeogenesis in cows [53,55]. As a confirmation of its important role in the core, genus *Succiniclasticum* was identified as key component of the epithelial microbiota in five of the sequencing projects that we analyzed. The importance of the reference database is also evident for other taxa. *Clostridiales* Family XIII *Incertae Sedis* and *Cardiobacteriaceae* were previously identified as key components of the epithelial microbiota in the three studies analyzing the results at the family level. However, our results do not confirm these findings, even if we lower the threshold for microbiota core detection to 75% of presence over all the samples. Other studies have identified *Prevotellaceae* and *Prevotella* as part of the epithelial microbiota as well as of the other ruminal microenvironments [21,23]. The relatively high prevalence of this taxon in our meta-analysis would suggest an important role played by these microbes in epimural community, as well as in the other niches of the rumen [46]. Bacteria belonging to the family *Prevotellaceae* are known to be able to process monosaccharides and polysaccharides, peptides, and proteins, and are usually enriched in high-grain diets [12,53,56]. Nevertheless, since *Prevotellaceae* were detected in studies with different feeding regimes, it is unlikely that diet was the factor responsible for the differences observed. It is possible that diverse species within this family prefer metabolic pathways that are also highly adaptable to environmental stressors such as low pH conditions [57].

*Methanobrevibacter* was identified in all the studies and in 83.7% of the samples. Methanogens are the dominant Archaea within the GI tract of animals [58] and, depending on the hypervariable region of the 16S rRNA gene, some primers also amplify archaeal gene sequences [59]. The majority of the studies included in this meta-analysis did not report the presence of archaeal features amongst their sequences. Methanogens have been previously reported to have preferably either a particle-adherent, protozoa-adherent, or a free-living lifestyle [23]. However, metatranscriptome sequencing also found *Methanobrevibacter* as a highly abundant methanogen in epimural microbiota, with a high number of transcripts belonging to glycan biosynthesis, methane metabolism, and glyoxylate and dicarboxylate metabolism [42]. A high prevalence in epithelial samples might indicate that their presence in the rumen and significance in this niche has been underestimated.

### 3.4. Canonical Correlation Analysis Discloses Predicted Functions of the Core Microbiota

**Figure 4:**
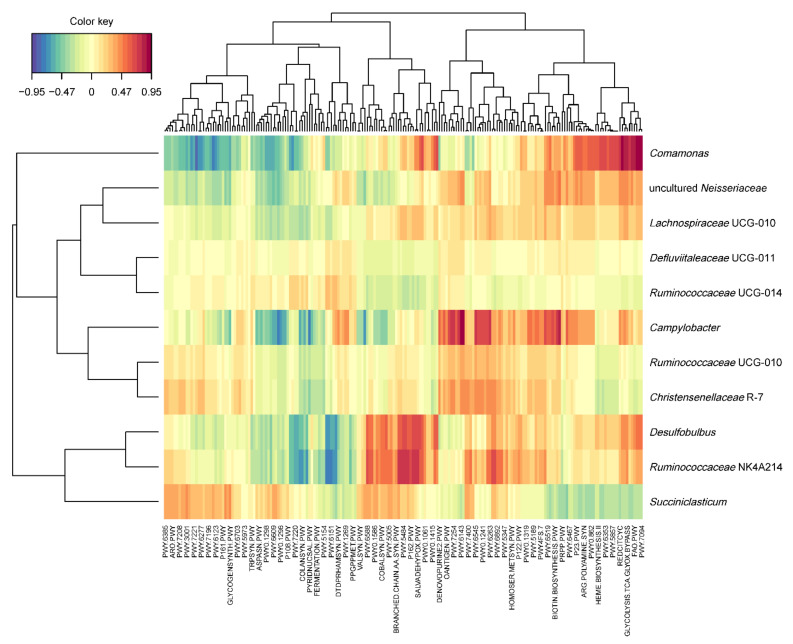
Clustered image map based on the similarity matrix obtained from the regularized canonical correlation analysis (rCCA) analysis of core genera identified in > 90% of the samples and putative pathways predicted with PICRUSt2.

Regularized canonical correlation analysis was conducted between the pathways found in all samples, and the core genus microbiota of the epithelium (Figure 4). Several metabolic pathways were identified associated with the *Ruminococcaceae* NK4A214 group, *Campylobacter, Comamonas,* and *Desulfobulbus* (|*r*| ≥ 0.7).

*Campylobacter* was mainly associated with pathways involved in cytosine-monophosphate-sugar biosynthesis (PWY-6143, *r* = 0.95), quinol and quinone biosynthesis (PWY-7373, *r* = 0.93; PWY-7371, *r* = 0.71; PWY-6263, *r* = 0.77), heme B biosynthesis (HEMESYN2-PWY, *r* = 0.81), tricarboxylic acid cycle (TCA) (TCA, *r* = 0.73; PWY-7254, *r* = 0.75; P42-PWY, *r* = 0.71), purine (PWY-841, *r* = 0.72; PWY-7228, *r* = 0.74) and pyrimidine (PWY-6545, *r* = 0.75) nucleotide biosynthesis, L-methionine biosynthesis (HSERMETANA-PWY, *r* = 0.7). Interestingly, *Campylobacter* is negatively correlated with starch degradation (PWY-6737, *r* = −0.72) and nicotinamide adenine dinucleotide (NAD) biosynthesis (PYRIDNUCSAL-PWY, *r* = −0.73). The function of *Campylobacter* in the rumen epithelium has for long been subject to debate. *Campylobacteraceae* are Gram-negative microaerophilic bacteria, which are usually associated with foodborne diseases in humans and diarrhea in pigs. Their presence in the cattle GI tract has been studied for years, as their commensal role in livestock is considered to be a reservoir for pathogen shedding [40,41]. Glutamate dehydrogenase and glutamine synthetase have been shown to be highly expressed by *Campylobacter* within the epimural microbiota [42]. This matches previous studies that have implied that *Campylobacteraceae* could be involved in protein metabolism. Quinol and quinones play a fundamental role in energy generating processes, as these compounds mediate respiratory electron transport. A periplasmic nitrate reductase napA and a thioredoxin reductase were previously found to be highly abundant in this genus [42]. This would be fundamental for anaerobic growth or auxiliary electron transport, allowing Campylobacter to anaerobically and aerobically succeed as a colonizer of the bovine gut, indicating a very likely role of Campylobacter in oxygen scavenging.

*Ruminococcaceae* NK4A214 group is positively correlated with quinol and quinone biosynthesis (PWY-7374, *r* = 0.76), L-glutamate degradation (P162-PWY, *r* = 0.71), *Bifidobacterium* shunt (P124-PWY, *r* = 0.78), purine nucleotide degradation (SALVADEHYPOX-PWY, *r* = 0.75, PWY-6608, *r* = 0.75), sulfur metabolism (PWY-5304, *r* = 0.74), glycolysis (PWY-5484, *r* = 0.74; GLYCOLYSIS, *r* = 0.72), and peptidoglycan biosynthesis (PWY-6471, *r* = 0.74) and is negatively correlated to folate biosynthesis (PWY-6612, *r* = −0.74) and S-adenosyl-L-methionine biosynthesis (PWY-6151, *r* = −0.74). *Comamonas* was found to be highly correlated with the glyoxylate bypass (TCA-GLYOX-BYPASS, *r* = 0.87; GLYOXYLATE-BYPASS, *r* = 0.90; GLYCOLYSIS-TCA-GLYOX-BYPASS, *r* = 0.85), fatty acid oxidation (FAO-PWY, *r* = 0.86) and biosynthesis (PWY-7094, *r* = 0.9), sulfate reduction (SO4ASSIM-PWY, *r* = 0.82) and assimilation (SULFATE-CYS-PWY, *r* = 0.71), proteinogenic amino acid degradation (TYRFUMCAT-PWY, *r* = 0.93; LEU-DEG2-PWY, *r* = 0.82, PWY-5345, *r* = 0.74), TCA cycle (P105-PWY, *r* = 0.82; REDCITCYC, *r* = 0.75), and purine nucleotide degradation (PWY-6353, *r* = 0.7). It is also negatively correlated to pyruvate fermentation to SCFA (PWY-5100, *r* = −0.81), L-threonine biosynthesis (THRESYN-PWY, *r* = −0.73) and purine nucleotide biosynthesis (PWY-6123, *r* = −0.71). Some studies suggest that *Comamonas* possibly intervenes in nitrate reducing and complex organic compound degrading processes [24] or that it may have an important role as an oxygen scavenger, similarly to *Desulfobulbus* [23]. The function of these families also appears to be fundamental for the processes of transformation of volatile fatty acids at the epimural level [60]. *Ruminococcaceae* NK4A214 group and *Comamonas* were positively correlated with sulfur metabolism, assimilation, and reduction pathways. *Ruminococcaceae* NK4A214 group was also negatively correlated with the biosynthesis of methionine, a sulfur-containing amino acid [61]. Microorganisms in the rumen reduce sulphate to sulfur, that can be included in organic compounds, such as proteins and essential amino acids [62]. Sulfate reduction has been previously assumed to have a negative effect on the rumen epithelium, given the toxicity of H_2_S [42]. *Comamonas*, on the other hand, was positively correlated with the TCA cycle, in which 5′-methylthioadenosine (MTA) is produced. This by-product can be used as source of methionine, sulfur, or purines [63]. In addition to their role in sulfur metabolism, our meta-analysis confirmed the role of *Comamonas* in nitrogen metabolism in the rumen. It was in fact positively correlated with proteinogenic amino acid degradation, and with purine nucleotide degradation. The latter pathway was also positively correlated with the *Ruminococcaceae* NK4A214 group. These findings suggest a fundamental function of both of these families in amino acidic and protein metabolism [64]. Our analysis did not confirm the role of *Desulfobulbus* in the metabolism of sulfur compounds, but we interestingly found other families being strongly correlated with such pathways. The core microbes identified in our meta-analysis were involved in amino acid metabolism to some extent, as previously suggested [42,65], highlighting a possible role of epimural microbes in nitrogen recycling, particularly the *Ruminococcaceae* NK4A214 group.

### 3.5. Factors Affecting the Epithelial Microbiota Composition and Structure

**Figure 5:**
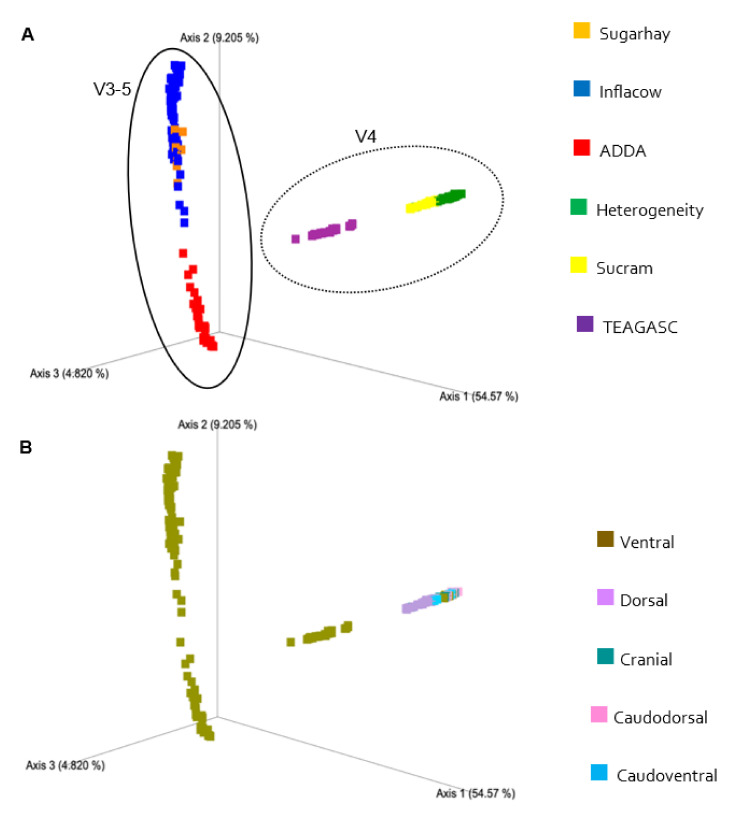
Principal-coordinate analysis of Bray-Curtis dissimilarities classified by study (**A**) and (**B**) biopsy location. The amount of variation explained by the principal coordinates is given in the axes (%). In (**A**), clustering seems to occur based on the hypervariable region used in the study.

**Figure 6:**
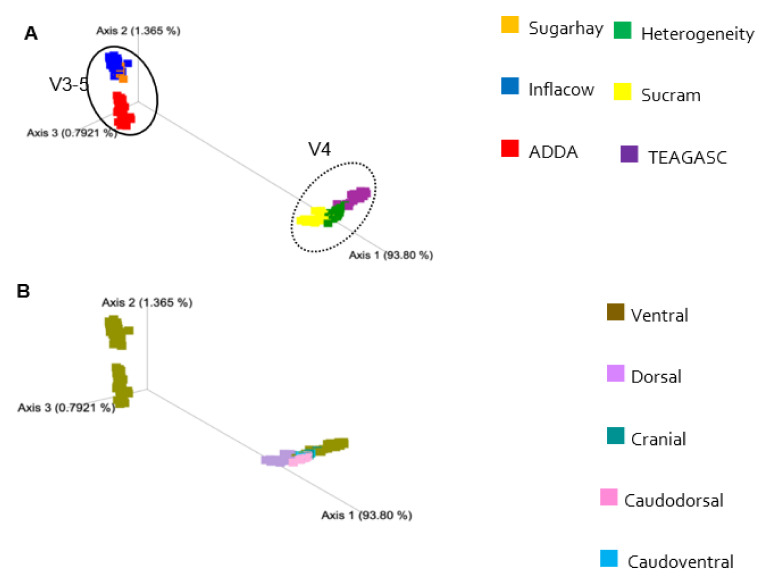
Principal-coordinate analysis of Weighted UniFrac distances classified by study (**A**,**B**) biopsy location. The amount of variation explained by the principal coordinates is given in the axes (%).

**Figure 7:**
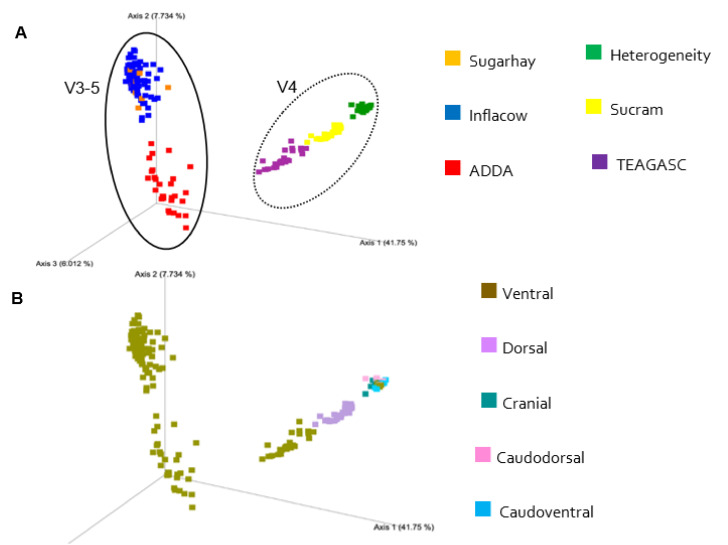
Principal-coordinate analysis of unweighted UniFrac distances classified by study (**A**,**B**) biopsy location. The amount of variation explained by the principal coordinates is given in the axes (%).

Comparisons between studies are not straightforward due to the sources of technical and biological variation inherent to each study. Differences in the laboratory methodologies employed, such as the DNA extraction method, the hypervariable region, PCR primers, sequencing platform, and bioinformatics pipeline all contributed to this variation [16]. Beta-diversity analyses showed unequivocal patterns of clustering mainly by hypervariable region sequenced. The PCoAs of Bray-Curtis dissimilarities (Figure 5) and both weighted (Figure 6) and unweighted UniFrac (Figure 7) distances also reveal a separation between studies, indicating a substantial similarity of taxonomic composition within sequencing projects. In the unweighted UniFrac plot, it is evident how each study represents a cluster, indicating a considerable qualitative difference in the microbiota composition between studies.

### 3.6. Technical Factors: Study Characteristics and Biases

**Figure 8:**
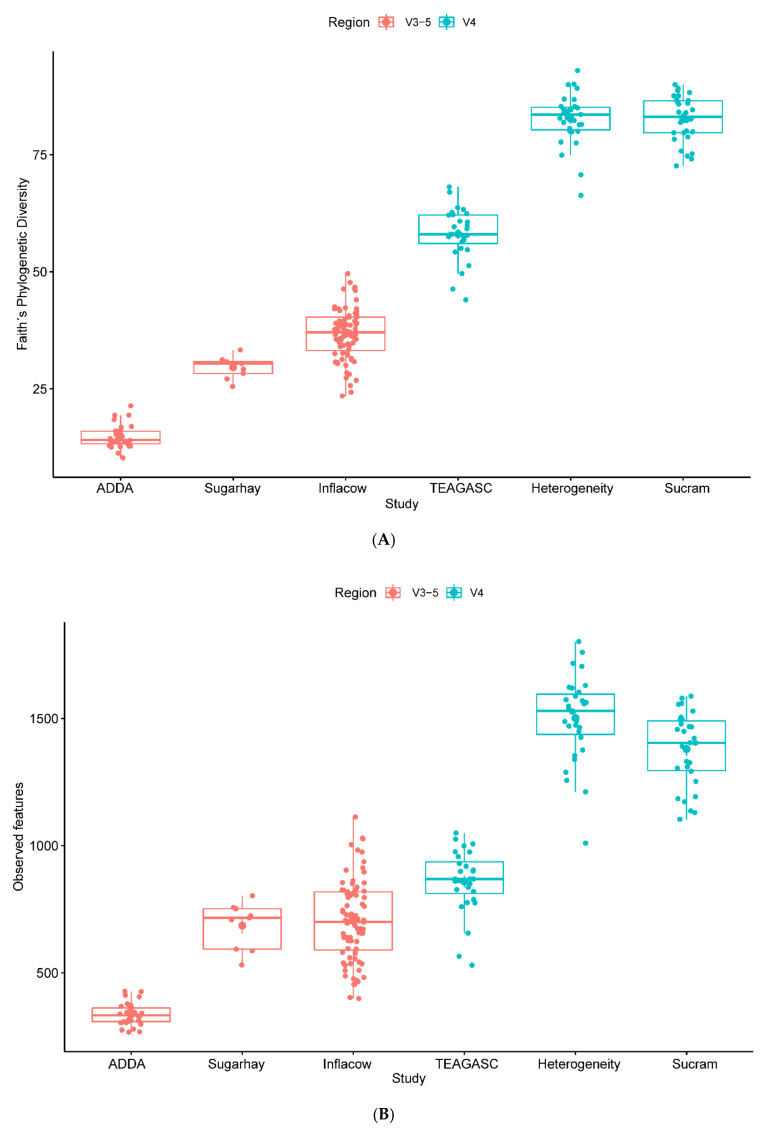

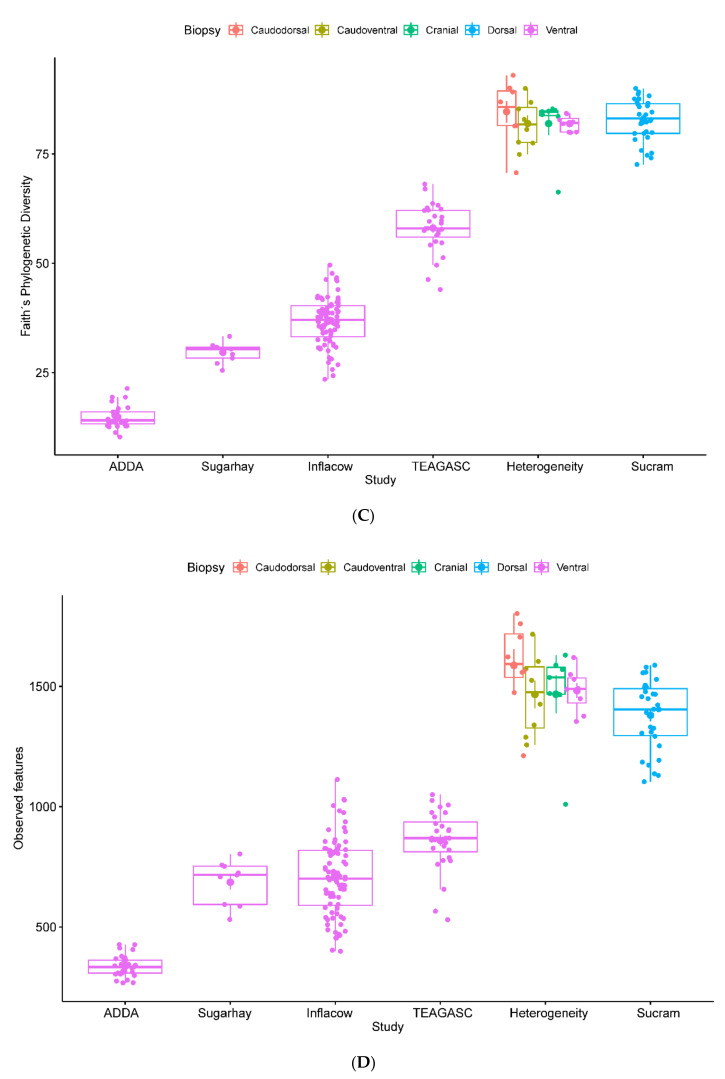
Faith phylogenetic diversity and richness estimator observed features measurements according to hypervariable region (**A**,**B**) and biopsy location (**C**,**D**), respectively.

All metadata categories were tested using PERMANOVA, ANOSIM, and PERMDISP (Table 3). The individual study had the largest effect in the inter-sample diversity, according to the PERMANOVA results on weighted (R^2^ = 0.97, *p* = 0.001) and unweighted (R^2^ = 0.70, *p* = 0.001) UniFrac distance matrices, whilst the individual variability seems to have the largest effect in the Bray-Curtis dissimilarity matrix (R^2^ = 0.65, *p* = 0.001). ANOSIM using the weighted UniFrac distance matrix revealed that the most significant separation is given by hypervariable region (R = 1.00, *p* = 0.001), followed by study (R = 0.94, *p* = 0.001), country (R = 0.94, *p* = 0.001), farm (R = 0.90, *p* = 0.001) and primer set (R = 0.89, *p* = 0.001). In the unweighted UniFrac, however, primer set (R = 0.95, *p* = 0.001) is the most important factor after the hypervariable region, while in Bray-Curtis dissimilarities, country (R = 0.94, *p* = 0.001) shows up as the second most important factor. However, almost all of these results can be associated with a possible dispersion effect, as PERMDISP was significant (*p* < 0.05) in most of the cases. Exceptions were found in the weighted UniFrac distances for country (*p* = 0.15) and in the Bray-Curtis dissimilarities for the hypervariable region (*p* = 0.98). Comparisons of alpha diversity measures revealed significant differences between V3–5 and V4 (*p* < 0.001). In fact, samples obtained through V3–5 amplicon sequencing had overall fewer of observed features and lower diversity than samples sequenced using the V4 hypervariable region (Figure 8A,B).

### 3.7. Influence of Hypervariable Region

In order to understand how the hypervariable region affected the microbiota composition, linear discriminant analysis was used to determine which genera were significantly over- and under-represented. Sixteen bacterial genera were identified as being affected by the bias introduced with the choice of the hypervariable region (Supplementary Figure S2). The methanogen *Methanobrevibacter* and the bacteria *Rikenellaceae* RC9 gut group and *Candidatus Saccharimonas* seem to be more prevalent in samples sequenced using the V4 or V3–4 than in samples using V3–5. The use of the region V3–5 led to an underrepresentation of sequences belonging to *Prevotella* 1, *Fibrobacter, Eubacterium nodatum group, Mogibacterium, Butyrivibrio* 2, and *Succinivibrionaceae* UCG-001, while incrementing the relative abundance of *Campylobacter,* the *Christensenellaceae* R7 group, the *Ruminococcaceae* NK4A214 group, *Ruminococcus* 1, *Desulfobulbus, Comamonas*, and an uncultured *Neisseriaceae*. The high relative abundance of *Epsilonbacteraeota* in the V3–5 studies is reflected at the family level, where the family *Campylobacteraceae* has a high relative abundance, especially in the projects Inflacow and Sugarhay. The detection of *Prevotellaceae* may be depending on the hypervariable region of choice: in fact, *Prevotella* is absent in all the Austrian studies, which employed the region V3–V5. This data show that future research looking at the identification of microbial shifts as biomarkers of disease should consider studies using the same hypervariable region to improve comparability.

### 3.8. Biological Factors: Animal and Biopsy

Several studies on the structure of the epithelium microbiome of cattle have focused on the influence of diet [9,10,18,20,24], or feed additives [18,19,22], but fewer have focused on the heterogeneity among ruminal locations [21]. The number of animals sampled varied greatly among studies. Some studies only sampled the same animal once, while other studies included several time-points (longitudinal studies) under different experimental conditions. In the weighted UniFrac distance matrix, individual variability is as significant as the study per se (R^2^ = 0.97, *p* = 0.001). The second most important factor is the biopsy location according to ANOSIM (R = 0.49, *p* = 0.001), which is again consistent with the results obtained for the other matrices. The most sampled ruminal location was the ventral sac (84% of the samples), followed by the dorsal sac (9% of the samples). Only one project included samples from different ruminal locations (ventral, cranial, caudodorsal, and caudoventral blind sac). Comparisons of alpha diversity measures revealed significant differences between biopsy locations (*p* < 0.001). A pairwise Wilcoxon rank sum test on the number of observed features identified significant differences between caudodorsal and dorsal (*p* < 0.01), caudodorsal and ventral (*p* < 0.01), caudoventral and ventral (*p* < 0.01), cranial and ventral (*p* < 0.01), and dorsal and ventral (*p* < 0.01) ruminal regions. The only significant difference was found in community richness between caudodorsal and dorsal sac. The ventral fraction was distinct from all the other ruminal locations. No differences in diversity were found between ruminal locations, except for the ventral sac (*p* < 0.01). However, this bias seems to be again associated with the use of the V3–5 hypervariable region. Samples of the ventral sac sequenced using V4 seem to be comparable to the other ruminal locations (Figure 8C,D).

### 3.9. Predicted Functional Potential Varies across Ruminal Locations

The ruminal epithelium consists of papillae, which increase the surface area for the absorption of SCFA [66] but considerable functional variation exists across the spatial distribution of this tissue layer. To further investigate which functions are associated with the epithelial microbiota, PICRUSt2 and LEfSE were used to identify which pathways were enriched among the samples relatively to biopsy location (Supplementary Figure S2). Pathways associated with the caudodorsal region are mainly involved in degradation/utilization/assimilation, biosynthesis, and generation of precursor metabolites and energy. These pathways are involved in D-galacturonate degradation (GALACTUROCAT_PWY), carbohydrate biosynthesis (COLANSYN_PWY), fermentation to SCFAs (P108_PWY), guanosine-5′-diphosphate sugar biosynthesis (PWY_7323), starch (PWY_6737) and sugar (PWY_6507, PWY_7242) degradation, pyrimidine nucleotide biosynthesis (PWY_7199), and glycogen degradation (GLYCOCAT_PWY). Interestingly, pathways enriched in the dorsal area of the rumen are mainly associated with quinol and quinone biosynthesis (PWY_5897, PWY_5898, PWY_5899, PWY_5840, PWY_5838), metabolic regulator biosynthesis (PPGPPMET_PWY), and lipopolysaccharide biosynthesis (KDO_NAGLIPASYN_PWY). The ventral side of the rumen had two overexpressed pathways mainly associated with the generation of precursor metabolites and energy, such as the TCA cycle (P105_PWY) and aerobic respiration/electron transfer chains (PWY_3781). The ventral epithelium maintains a higher blood flow and is more papillated than the dorsal epithelium, being thus more implicated in nutrient absorption. Ruminal contractions and stratification of rumen contents are likely to affect dorsal and medial locations more than ventral sites [3]. Therefore, differences in specific metabolic functions within the different ruminal locations are very plausible.

### 3.10. A Closer Look into Austrian Samples

**Figure 9:**
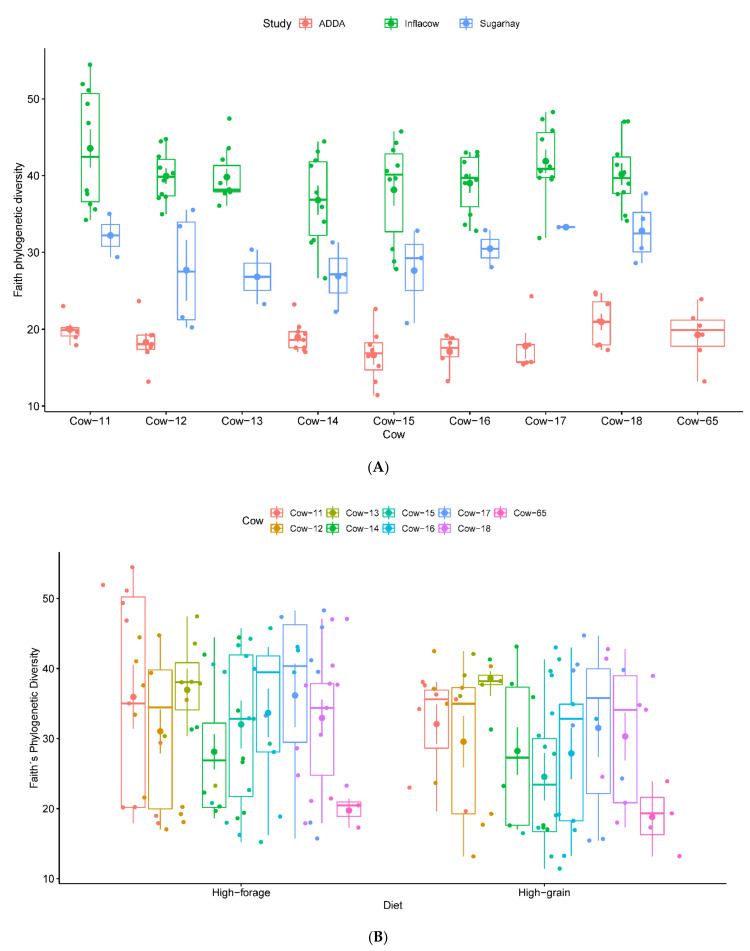
Phylogenetic diversity according to animal variability per each study (**A**) and diet per individual cow (**B**).

Samples from studies conducted in Austria (Projects Inflacow, ADDA, and Sugarhay) always used the same hypervariable region (V3–5) and biopsy location (ventral sac). Furthermore, the animals were housed in the same farm, allowing us to control the bias induced both by the farm and country. These samples were tested for the effect of the individual study, primer set, individual cow and diet. PERMANOVA on the weighted UniFrac distance matrix identified study (R^2^ = 0.37, *p* = 0.001), followed by primer (R^2^ = 0.22, *p* = 0.001) diet (R^2^ = 0.10, *p* = 0.001), and individual cow (R^2^ = 0.02, *p* = 0.015) as significant factors impacting the structure of the epimural microbiota. This is consistent with the permutational tests performed on the unweighted UniFrac distance matrix and the Bray-Curtis dissimilarity matrix. ANOSIM on the weighted UniFrac distance matrix shows that individual cow is not statistically significant (R = 0.02, *p* = 0.10). The composition of the GI tract microbiome, when geography and experimental setup is similar, is mainly affected by diet, despite the recognized influence of age, breed, environment and host genetics [12,46,67]. In fact, if we remove the sources of technical variation (study and primer), diet (*p* = 0.03) has a stronger effect than individual variability (Figure 9).

## 4. Conclusions

The richness, diversity, and complexity of the rumen ecosystem makes it a well-studied microbial community. However, despite having been clearly identified as a unique component nearly 40 years ago, the epimural microbiome remains the least understood of the rumen niches. The re-analysis of 11 studies showed that a core set of microbes are associated with the rumen epithelium despite differences in experimental factors and methodologies. Sampling sites within the rumen showed variation in microbial communities and their predicted metabolism. However, this variation was compounded by the methods used for sequencing and bioinformatics analysis. Specifically, the use of a consistent variable region will be critical for future evaluations of the epimural community, since this factor alone greatly impacts diversity measurements and the comparability of datasets. Therefore, bioinformatics standards need to be addressed to ensure the accurate analysis of future data. Only by advancing our understanding of the microbes, their metabolism, and the critical factors affecting these components, can we apply precision methods for stabilizing rumen and animal health.

## Figures and Tables

**Table 1:** Studies included in the meta-analysis. N stands for the total number of sequences retrieved in each study.

Project	Country	Animal	Breed	Biopsy Location	Sampling	N	Accession Numbers	References
Inflacow	Austria	Cow	Holstein-Friesian	Ventral sac	8 rumen-cannulated animals, longitudinal	80	PRJEB12642, PRJEB9353, SRP158246	[9,10,17]
ADDA	Austria	Cow	Holstein-Friesian	Ventral sac	8 rumen-cannulated animals, longitudinal	96	PRJEB29866, PRJEB33839	[18,19]
Sugarhay	Austria	Cow	Holstein-Friesian	Ventral sac	8 rumen-cannulated animals, longitudinal	32	PRJEB22005	[20]
Heterogeneity	USA	Cow	Holstein-Friesian	Cranial, ventral, caudodorsal and caudoventral blind sac	8 rumen-cannulated animals, one time-point	31	PRJNA562284	[21]
Sucram	USA	Cow	Holstein-Friesian	Dorsal sac	5 rumen-cannulated animals, longitudinal	30	PRJNA554894	[22] (preprint)
RumEnv	Belgium	Cow	Holstein-Friesian	Ventral sac	4 rumen-cannulated animals, one time-point	4	PRJN318319, SRP074884	[23]
Spring	Germany	Cow	Holstein-Friesian	Ventral sac	10 rumen-cannulated animals, longitudinal	30	PRJEB19414	[24]
TEAGASC	Ireland	Steer	Charolais, Holstein-Friesian	Ventral sac	29 animals post mortem, one time-point	29	PRJNA484585	Unpublished

**Table 2:** Detailed experimental characteristics of the studies included in the meta-analysis.

Project	16S Region	Sequencer	Extraction Method	Amplicon	PCR Primer	References
Inflacow	V3–5	Illumina MiSeq	PowerSoil Kit (MO BIO)	2 × 300 bp	341F (5′CCTACGGGRSGCAGCAG3′); 909R (5′TTTCAGYCTTGCGRCCGTAC3′)	[9,10,17]
ADDA	V3–5	Illumina MiSeq	Pre-lysis with heating, chemical lysis and bead beating + Power-Soil Kit (MO BIO)	2 × 300 bp	357F (5′CCTACGGGAGGCAGCAG3′); 926R (5′CCGTCAATTCMTTTRAGT3′)	[18,19]
Sugarhay	V3–5	Illumina MiSeq	Pre-lysis with heating, chemical lysis and bead beating + Power-Soil Kit (MO BIO)	2 × 300 bp	357F (5′CCTACGGGAGGCAGCAG3′); 926R (5′CCGTCAATTCMTTTRAGT3′)	[20]
Heterogeneity	V4	Illumina MiSeq	Bead beating + hot phenol method	2 × 250 bp	515F (5′GTGCCAGCMGCCGCGGTAA3′); 806R (5′GGACTACHVGGGTWTCTAAT3′)	[21]
Sucram	V4	Illumina MiSeq	Bead beating + DNeasy Powerlyzer PowerSoil Kit (Qiagen)	2 × 250 bp	515F (5′GTGYCAGCMGCCGCGGTAA3′); 806R (5′GGACTACNVGGGTWTCTAAT3′)	[22] (preprint)
RumEnv	V3–4	Illumina MiSeq	Bead beating + column (RBB + C) protocol	2 × 300 bp	344F (5′CCTACGGGNGGCWGCAG3′); 806R (5′GACTACHVGGGTATCTAATCC3′)	[23]
Spring	V4	Illumina MiSeq	peqGold Tissue-Kit	2 × 250 bp	519F (5′CAGCMGCCGCGGTAA3′); 802R (5′TACNVGGGTATCTAATCC3′)	[24]
TEAGASC	V4	Illumina MiSeq	Bead beating + column (RBB + C) protocol	2 × 250 bp	515F (5′GTGCCAGCMGCCGCGGTAA3′); 806R (5′GGACTACHVGGGTWTCTAAT3′)	Unpublished

**Table 3:** Metadata categories affecting the epimural microbiota community structure assessed using permutational multivariate analysis of variance (PERMANOVA), permutational analysis of multivariate dispersions (PERMDISP), and analysis of similarities (ANOSIM).

Parameter	Weighted UniFrac					Unweighted UniFrac					Bray-Curtis				
	PERMANOVA		PERMDISP	ANOSIM		PERMANOVA		PERMDISP	ANOSIM		PERMANOVA		PERMDISP	ANOSIM	
	R^2^	*p*	*p*	R	*p*	R^2^	*p*	*p*	R	*p*	R^2^	*p*	*p*	R	*p*
Study	0.97	0.00	0.02	0.94	0.00	0.70	0.00	0.00	0.93	0.00	0.59	0.00	0.00	0.92	0.00
Country	0.95	0.00	0.15	0.94	0.00	0.59	0.00	0.00	0.91	0.00	0.47	0.00	0.00	0.94	0.00
Farm	0.95	0.00	0.00	0.90	0.00	0.61	0.00	0.00	0.82	0.00	0.51	0.00	0.00	0.88	0.00
Gene region	0.94	0.00	0.03	1.00	0.00	0.54	0.00	0.00	1.00	0.00	0.41	0.00	0.98	1.00	0.00
Primer	0.95	0.00	0.00	0.89	0.00	0.61	0.00	0.00	0.95	0.00	0.47	0.00	0.00	0.89	0.00
Individual	0.97	0.00	0.00	0.59	0.00	0.69	0.00	0.00	0.54	0.00	0.65	0.00	0.08	0.59	0.00
Gender	0.19	0.00	0.00	0.20	0.00	0.14	0.00	0.00	0.09	0.00	0.11	0.00	0.00	0.32	0.00
Biopsy location	0.45	0.00	0.01	0.49	0.00	0.29	0.00	0.01	0.46	0.00	0.27	0.00	0.00	0.39	0.00

## Data Availability

No new data were created or analyzed in this study. Data sharing is not applicable to this article.

## Supplementary Materials

The following are available online at https://www.mdpi.com/2076-2607/9/2/342/s1, Figure S1. Abundance histograms of features at the genus level identified by LEfSe as differentially abundant according to the target region of the 16S rRNA gene, Figure S2. Metabolic pathways identified by LEfSe as differentially abundant according to the biopsy location, Table S1. Bioinformatics processing of the sequences included in the meta-analysis, Table S2 MetaCyc pathways and BioCyc IDs.

## References

[1] Malmuthuge N., Guan L.L. (2017). Understanding host-microbial interactions in rumen: Searching the best opportunity for microbiota manipulation. J. Anim. Sci. Biotechnol..

[2] Liu J.H., Zhang M.L., Zhang R.Y., Zhu W.Y., Mao S.Y. (2016). Comparative studies of the composition of bacterial microbiota associated with the ruminal content, ruminal epithelium and in the faeces of lactating dairy cows. Microb. Biotechnol..

[3] Millen D., De Beni Arrigoni M., Lauritano Pacheco R.D. (2016). Rumenology.

[4] Hungate R.E. (1966). The Rumen and Its Microbes.

[5] Li M., Zhou M., Adamowicz E., Basarab J.A., Guan L.L. (2012). Characterization of bovine ruminal epithelial bacterial communities using 16S rRNA sequencing, PCR-DGGE, and qRT-PCR analysis. Vet. Microbiol..

[6] Cheng K.J., Bailey C.B., Hironaka R., Costerton J.W. (1979). A technique for depletion of bacteria adherent to the epithelium of the bovine rumen. Can. J. Anim. Sci..

[7] Cheng K.J., McCowan R.P., Costerton J.W. (1979). Adherent epithelial bacteria in ruminants and their roles in digestive tract function. Am. J. Clin. Nutr..

[8] Jami E., Israel A., Kotser A., Mizrahi I. (2013). Exploring the bovine rumen bacterial community from birth to adulthood. ISME J..

[9] Wetzels S.U., Mann E., Pourazad P., Qumar M., Pinior B., Metzler-Zebeli B.U., Wagner M., Schmitz-Esser S., Zebeli Q. (2017). Epimural bacterial community structure in the rumen of Holstein cows with different responses to a long-term subacute ruminal acidosis diet challenge. J. Dairy Sci..

[10] Wetzels S.U., Emann E., Metzler-Zebeli B.U., Epourazad P., Equmar M., Eklevenhusen F., Epinior B., Ewagner M., Ezebeli Q., Schmitz-Esser S. (2016). Epimural indicator phylotypes of transiently-induced subacute ruminal acidosis in dairy cattle. Front. Microbiol..

[11] Marchesi J.R., Ravel J. (2015). The vocabulary of microbiome research: A proposal. Microbiome.

[12] Henderson G., Cox F., Ganesh S., Jonker A., Young W., Janssen P.H., Abecia L., Angarita E., Aravena P., Global Rumen Census Collaborators (2015). Rumen microbial community composition varies with diet and host, but a core microbiome is found across a wide geographical range. Sci. Rep..

[13] Auffret M.D., Dewhurst R.J., Duthie C.-A., Rooke J.A., Wallace R.J., Freeman T.C., Stewart R.D., Watson M., Roehe R. (2017). The rumen microbiome as a reservoir of antimicrobial resistance and pathogenicity genes is directly affected by diet in beef cattle. Microbiome.

[14] Zebeli Q., Metzler-Zebeli B.U. (2012). Interplay between rumen digestive disorders and diet-induced inflammation in dairy cattle. Res. Vet. Sci..

[15] Waite D.W., Taylor M.W. (2014). Characterizing the avian gut microbiota: Membership, driving influences, and potential function. Front. Microbiol..

[16] Brooks J.P., Edwards D.J., Rivera M.C., Fettweis J.M., Serrano M.G., Reris R.A., Sheth N.U., Huang B., Jefferson K.K., Buck G.A. (2015). The truth about metagenomics: Quantifying and counteracting bias in 16S rRNA studies Ecological and evolutionary microbiology. BMC Microbiol..

[17] Petri R.M., Wetzels S.U., Qumar M., Khiaosa-ard R., Zebeli Q. (2019). Adaptive responses in short-chain fatty acid absorption, gene expression, and bacterial community of the bovine rumen epithelium recovered from a continuous or transient high-grain feeding. J. Dairy Sci..

[18] Petri R.M., Neubauer V., Humer E., Kröger I., Reisinger N., Zebeli Q. (2020). Feed Additives Differentially Impact the Epimural Microbiota and Host Epithelial Gene Expression of the Bovine Rumen Fed Diets Rich in Concentrates. Front. Microbiol..

[19] Neubauer V., Humer E., Mann E., Kröger I., Reisinger N., Wagner M., Zebeli Q., Petri R. (2019). Effects clay mineral supplementation on particle-associated and epimural microbiota, and gene expression in the rumen of cows fed high-concentrate diet. Anaerobe.

[20] Petri R.M., Kleefisch M.T., Metzler-Zebeli B.U., Zebeli Q., Klevenhusen F. (2018). Changes in the rumen epithelial microbiota of cattle and host gene expression in response to alterations in dietary carbohydrate composition. Appl. Environ. Microbiol..

[21] Sbardellati D.L., Fischer A., Cox M.S., Li W., Kalscheur K.F., Suen G. (2020). The bovine epimural microbiota displays compositional and structural heterogeneity across different ruminal locations. J. Dairy Sci..

[22] Koester L.R., Anderson C.J., Cortes B.W., Lyte M. (2020). Influence of the artificial sodium saccharin sweetener Sucram^®^ on the microbial community composition in the rumen content and attached to the rumen epithelium in dairy cattle: A pilot study. BioRxiv.

[23] De Mulder T., Goossens K., Peiren N., Vandaele L., Haegeman A., De Tender C., Ruttink T., Van De Wiele T., De Campeneere S. (2017). Exploring the methanogen and bacterial communities of rumen environments: Solid adherent, fluid and epimural. FEMS Microbiol. Ecol..

[24] Schären M., Kiri K., Riede S., Gardener M., Meyer U., Hummel J., Urich T., Breves G., Dänicke S. (2017). Alterations in the rumen liquid-, particle- and epithelium-associated microbiota of dairy cows during the transition from a silage- and concentrate-based ration to pasture in spring. Front. Microbiol..

[25] (2015). FastQC: A Quality Control Tool for High Throughput Sequence. Http://www.bioinformatics.babraham.ac.uk/projects/fastqc/.

[26] Lindgreen S. (2012). AdapterRemoval: Easy cleaning of next-generation sequencing reads. BMC Res. Notes.

[27] Bolyen E., Rideout J.R., Dillon M.R., Bokulich N.A., Abnet C.C., Al-Ghalith G.A., Alexander H., Alm E.J., Arumugam M., Asnicar F. (2019). Reproducible, interactive, scalable and extensible microbiome data science using QIIME 2. Nat. Biotechnol..

[28] Rognes T., Flouri T., Nichols B., Quince C., Mahé F. (2016). VSEARCH: A versatile open source tool for metagenomics. PeerJ.

[29] Amir A., McDonald D., Navas-Molina J.A., Kopylova E., Morton J.T., Xu Z.Z., Kightley E.P., Thompson L.R., Hyde E.R., Gonzalez A. (2017). Deblur Rapidly Resolves Single-Nucleotide Community Sequence Patterns. mSystems.

[30] Quast C., Pruesse E., Yilmaz P., Gerken J., Schweer T., Yarza P., Peplies J., Glöckner F.O. (2013). The SILVA ribosomal RNA gene database project: Improved data processing and web-based tools. Nucleic Acids Res..

[31] Caporaso J.G., Kuczynski J., Stombaugh J., Bittinger K., Bushman F.D., Costello E.K., Fierer N., Peña A.G., Goodrich J.K., Gordon J.I. (2010). QIIME Allows Analysis of High-Throughput Community Sequencing data. Nat. Methods.

[32] Douglas G.M., Maffei V.J., Zaneveld J., Yurgel S.N., Brown J.R., Taylor C.M., Huttenhower C., Langille M.G.I. (2019). PICRUSt2: An improved and extensible approach for metagenome inference. BioRxiv.

[33] Caspi R., Altman T., Billington R., Dreher K., Foerster H., Fulcher C.A., Holland T.A., Keseler I.M., Kothari A., Kubo A. (2014). The MetaCyc database of metabolic pathways and enzymes and the BioCyc collection of Pathway/Genome Databases. Nucleic Acids Res..

[34] R Core Team (2012). R: A Language and Environment for Statistical Computing.

[35] Benjamini Y., Hochberg Y. (1995). Controlling the False Discovery Rate: A Practical and Powerful Approach to Multiple Testing. J. R. Stat. Soc. Ser. B Methodol..

[36] Oksanen J., Blanchet F.G., Kindt R., Legendre P., Minchin P.R., O’hara R., Simpson G.L., Solymos P., Stevens M.H.H. (2013). Package ‘Vegan’. Community Ecology Package, Version 2. https://cran.r-project.org/package=vegan.

[37] Segata N., Izard J., Waldron L., Gevers D., Miropolsky L., Garrett W.S., Huttenhower C. (2011). Metagenomic biomarker discovery and explanation. Genome Biol..

[38] Waite D.W., VanWonterghem I., Rinke C., Parks D.H., Zhang Y., Takai K., Sievert S.M., Simon J., Campbell B.J., Hanson T.E. (2017). Comparative genomic analysis of the class Epsilonproteobacteria and proposed reclassification to epsilonbacteraeota (phyl. nov.). Front. Microbiol..

[39] Waite D.W., Chuvochine M.S., Hugenholz P., Trujillo M.E., Dedysh S., DeVos P., Hedlund B., Kämpfer P., Rainey F.A., Whitman W.B. (2019). Road Map of the Phylum Campylobacterota. Bergey’s Manual of Systematics of Archaea and Bacteria.

[40] Sproston E.L., Ogden I.D., Macrae M., Dallas J.F., Sheppard S.K., Cody A.J., Colles F.M., Wilson M., Forbes K.J., Strachan N.J.C. (2011). Temporal variation and host association in the Campylobacter population in a longitudinal ruminant farm study. Appl. Environ. Microbiol..

[41] Besser T.E., Lejeune J.T., Rice D.H., Berg J., Stilborn R.P., Kaya K., Bae W., Hancock D.D. (2005). Increasing prevalence of Campylobacter jejuni in feedlot cattle through the feeding period. Appl. Environ. Microbiol..

[42] Mann E., Wetzels S.U., Wagner M., Zebeli Q., Schmitz-Esser S. (2018). Metatranscriptome sequencing reveals insights into the gene expression and functional potential of rumen wall bacteria. Front. Microbiol..

[43] Liu G., Tang C.M., Exley R.M. (2015). Non-pathogenic neisseria: Members of an abundant, multi-habitat, diverse genus. Microbiology.

[44] Jin D., Zhao S., Wang P., Zheng N., Bu D., Beckers Y., Wang J. (2016). Insights into abundant rumen ureolytic bacterial community using rumen simulation system. Front. Microbiol..

[45] Bennett J.S., Jolley K.A., Maiden M.C.J. (2013). Genome sequence analyses show that Neisseria oralis is the same species as ‘Neisseria mucosa var. Heidelbergensis’. Int. J. Syst. Evol. Microbiol..

[46] Holman D.B., Gzyl K.E. (2019). A meta-analysis of the bovine gastrointestinal tract microbiota. FEMS Microbiol. Ecol..

[47] Henderson G., Yilmaz P., Kumar S., Forster R.J., Kelly W.J., Leahy S.C., Guan L.L., Janssen P.H. (2019). Improved taxonomic assignment of rumen bacterial 16S rRNA sequences using a revised SILVA taxonomic framework. PeerJ.

[48] Kenters N., Henderson G., Jeyanathan J., Kittelmann S., Janssen P.H. (2011). Isolation of previously uncultured rumen bacteria by dilution to extinction using a new liquid culture medium. J. Microbiol. Methods.

[49] Compant S., Nowak J., Coenye T., Clément C., Barka E.A. (2008). Diversity and occurrence of *Burkholderia* spp. in the natural environment. FEMS Microbiol. Rev..

[50] Voordouw G. (1995). The genus Desulfovibrio: The centennial. Appl. Environ. Microbiol..

[51] Steger J.L., Vincent C., Ballard J.D., Krumholz L.R. (2002). Desulfovibrio sp. genes involved in the respiration of sulfate during metabolism of hydrogen and lactate. Appl. Environ. Microbiol..

[52] Vacca M., Celano G., Calabrese F.M., Portincasa P., Gobbetti M., de Angelis M. (2020). The Controversial Role of Human Gut Lachnospiraceae. Microorganisms.

[53] Deusch S., Camarinha-Silva A., Conrad J., Beifuss U., Rodehutscord M., Seifert J. (2017). A structural and functional elucidation of the rumen microbiome influenced by various diets and microenvironments. Front. Microbiol..

[54] Zeng H., Guo C., Sun D., Seddik H.E., Mao S. (2019). The ruminal microbiome and metabolome alterations associated with diet-induced milk fat depression in dairy cows. Metabolites.

[55] Van Gylswyk N.O. (1995). *Succiniclasticum ruminis* gen. nov., sp. nov., a ruminal bacterium converting succinate to propionate as the sole energy-yielding mechanism. Int. J. Syst. Bacteriol..

[56] Krause D.O., Denman S.E., Mackie R.I., Morrison M., Rae A.L., Attwood G.T., McSweeney C.S. (2003). Opportunities to improve fiber degradation in the rumen: Microbiology, ecology, and genomics. FEMS Microbiol. Rev..

[57] Petri R.M., Schwaiger T., Penner G.B., Beauchemin K.A., Forster R.J., McKinnon J.J., Bie X. (2013). Changes in the rumen epimural bacterial diversity of beef cattle as affected by diet and induced ruminal acidosis. Appl. Environ. Microbiol..

[58] Janssen P.H., Kirs M. (2008). Structure of the archaeal community of the rumen. Appl. Environ. Microbiol..

[59] Holman D.B., Brunelle B.W., Trachsel J., Allen H.K. (2017). Meta-analysis To Define a Core Microbiota in the Swine Gut. mSystems.

[60] Bickhart D.M., Weimer P.J. (2018). Symposium review: Host–rumen microbe interactions may be leveraged to improve the productivity of dairy cows. J. Dairy Sci..

[61] Brosnan J., Brosnan M. (2006). 5th Amino Acid Assessment Workshop—The Sulfur-Containing Amino Acids: An Overview. J. Nutr..

[62] Kandylis K. (1984). Toxicology of Sulfur in Ruminants: Review. J. Dairy Sci..

[63] Albers E. (2009). Metabolic characteristics and importance of the universal methionine salvage pathway recycling methionine from 5′-methylthioadenosine. IUBMB Life.

[64] Fujihara T., Shem M.N. (2011). Metabolism of microbial nitrogen in ruminants with special reference to nucleic acids. Anim. Sci. J..

[65] Mao S., Zhang M., Liu J., Zhu W. (2015). Characterising the bacterial microbiota across the gastrointestinal tracts of dairy cattle: Membership and potential function. Sci. Rep..

[66] Dieho K., Bannink A., Geurts I.A.L., Schonewille J.T., Gort G., Dijkstra J. (2016). Morphological adaptation of rumen papillae during the dry period and early lactation as affected by rate of increase of concentrate allowance. J. Dairy Sci..

[67] Weimer P.J. (2015). Redundancy, resilience, and host specificity of the ruminal microbiota: Implications for engineering improved ruminal fermentations. Front. Microbiol..

