# Supplementary material for: Unveiling the Bovine Epimural Microbiota Composition and Putative Function
Source: Microorganisms. 2021 Feb 9;9(2):342. doi: 10.3390/microorganisms9020342 (PMC7915655; doi:10.3390/microorganisms9020342)

**Table S1. Bioinformatics processing of the sequences included in the meta-analysis.**

| Project | Input sequences | FASTQC  AdapterRemoval | VSEARCH  *--p-maxee 2* | q-score joined | | deblur | | | References |
| --- | --- | --- | --- | --- | --- | --- | --- | --- | --- |
|  |  |  |  | *--p-min-quality 20* | **% reads** | *--p-len 200* | **% reads** | Number of features |  |
| Inflacow | 10,475,806 | 10,475,804 | 5,617,709 | 4,903,833 | **46.8** | 3,508,031 | **33.5** | 11,346 | [9, 10, 17] |
| ADDA | 1,839,534 | 1,839,534 | 1,328,441* | 1,218,055** | **66.2** | 596,849 | **32.4** | 4,142 | [18, 19] |
| Sugarhay | 489,765 | 489,765 | 372,974* | 348,469** | **71.2** | 186,187 | **38.0** | 3,555 | [20] |
| Heterogeneity | 2,886,928 | 2,886,928 | 2,422,470 | 2,422,307 | **83.9** | 1,763,453 | **61.1** | 11,418 | [21] |
| Sucram | 5,032,179 | 5,032,179 | 3,393,235 | 3,392,066 | **67.4** | 2,144,061 | **42.6** | 30,747 | [22] (preprint) |
| RumEnv | 293,100 | 293,094 | 18,988 | 18,974 | **6.5** | 10,032 | **3.4** | 967 | [23] |
| Spring | 149,531 | 149,531 | 107,095 | 106,555 | **71.3** | 55,476 | **37.1** | 1,087 | [24] |
| TEAGASC | 1,786,328 | 1,786,328 | 1,613,411 | 1,610,892 | **90.2** | 1,264,121 | **70.8** | 6,877 | Unpublished |

(*)Parameter *--p-maxee* not used;

(**)Parameter *--p-min-quality 10* used instead of *20.*

**Figure S1. Abundance histograms of features at the genus level identified by LEfSe as differentially abundant according to the target region of the 16S rRNA gene.**

*Methanobrevibacter*


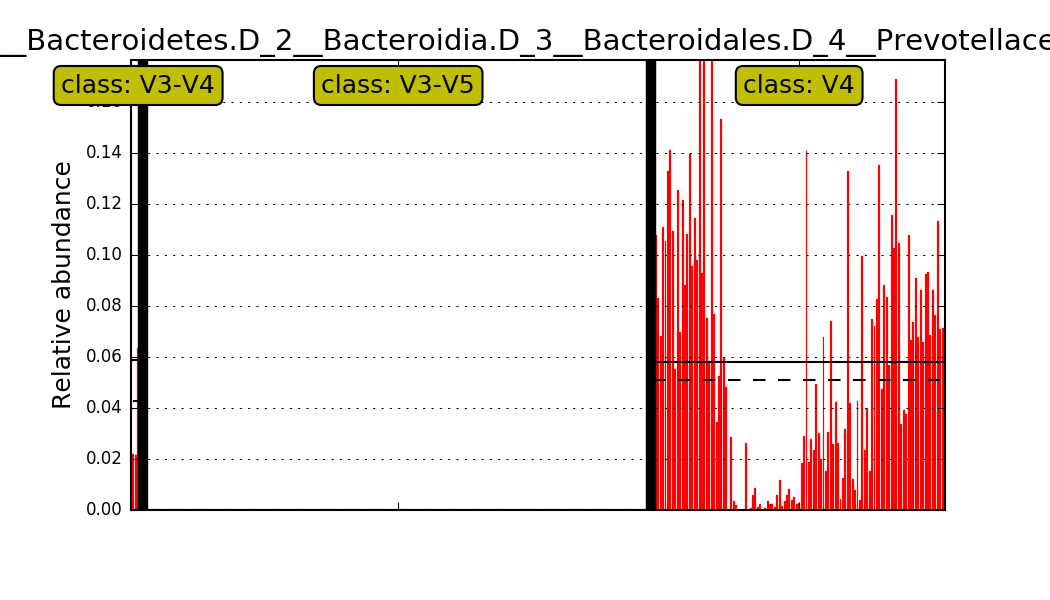


*Prevotella* 1


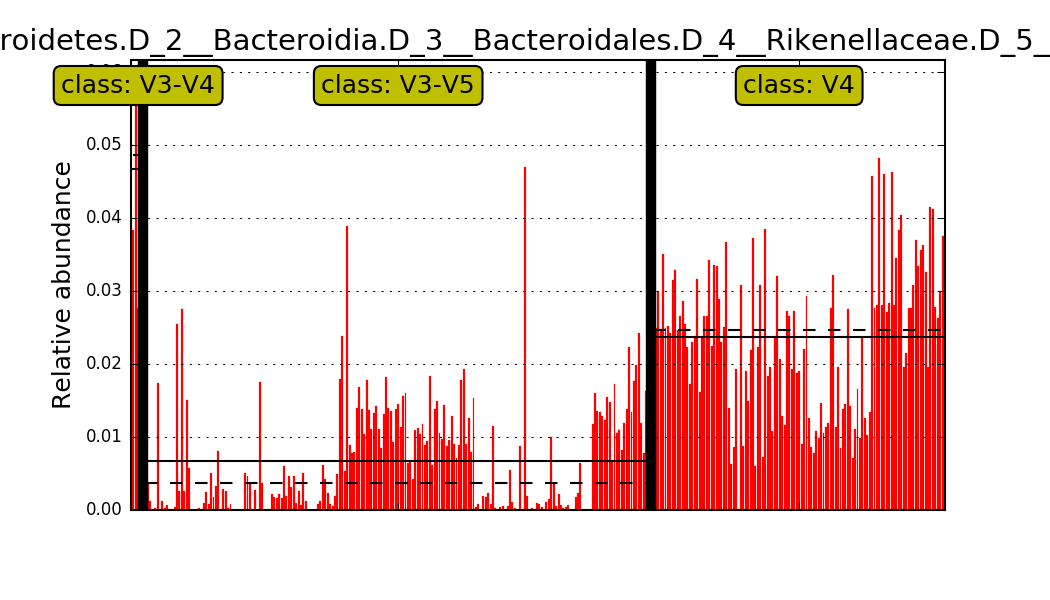


*Rikenellaceae* RC9 gut group


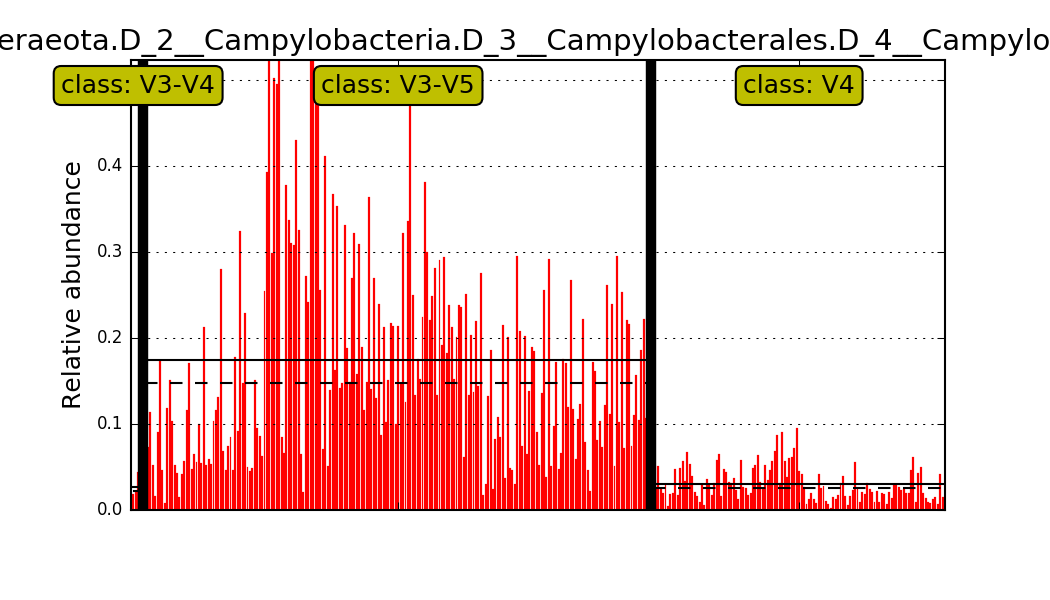

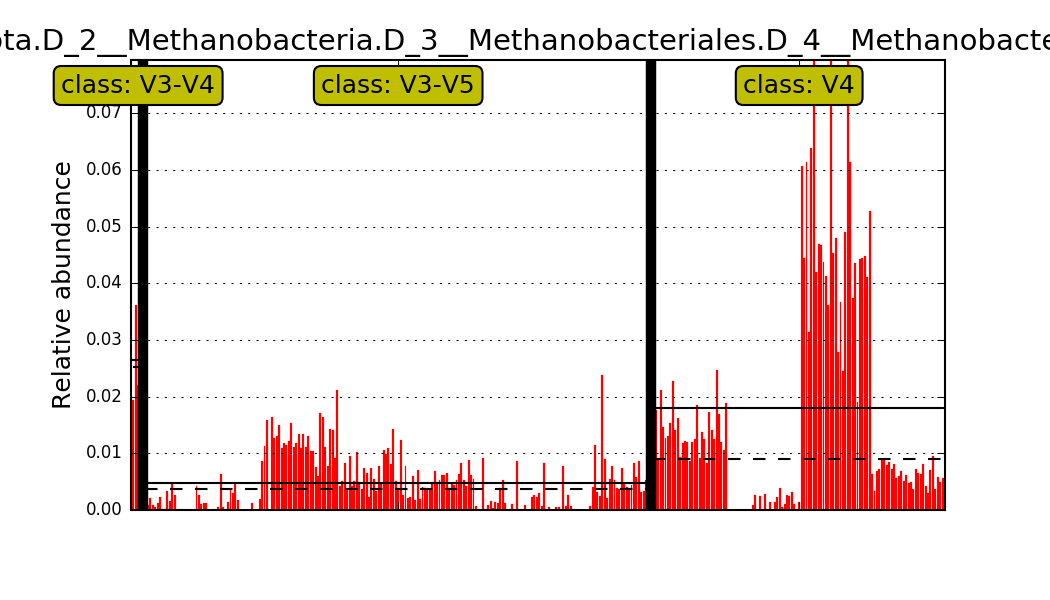


*Campylobacter*


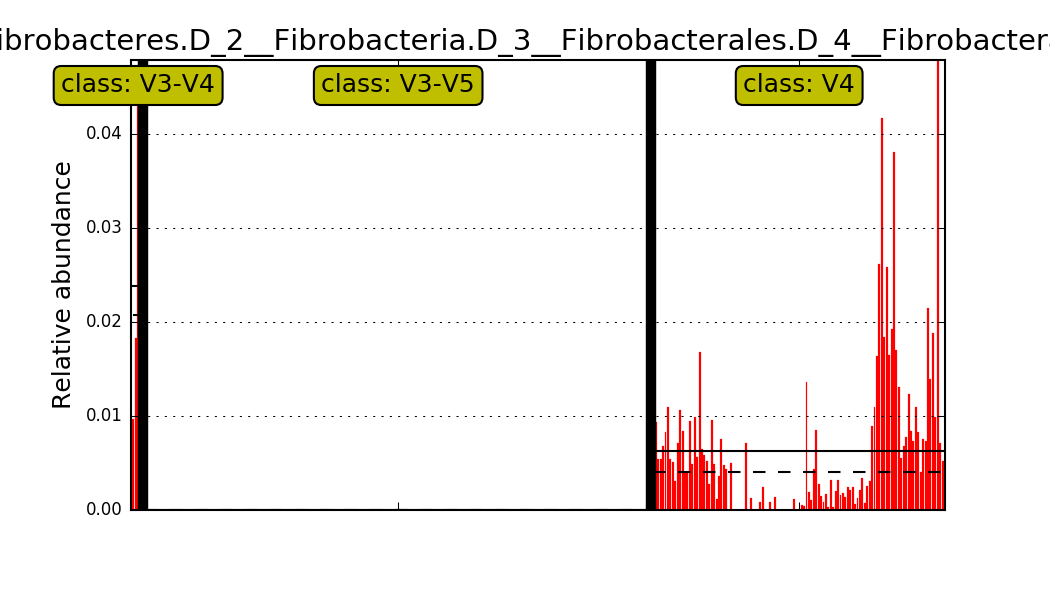


*Fibrobacter*


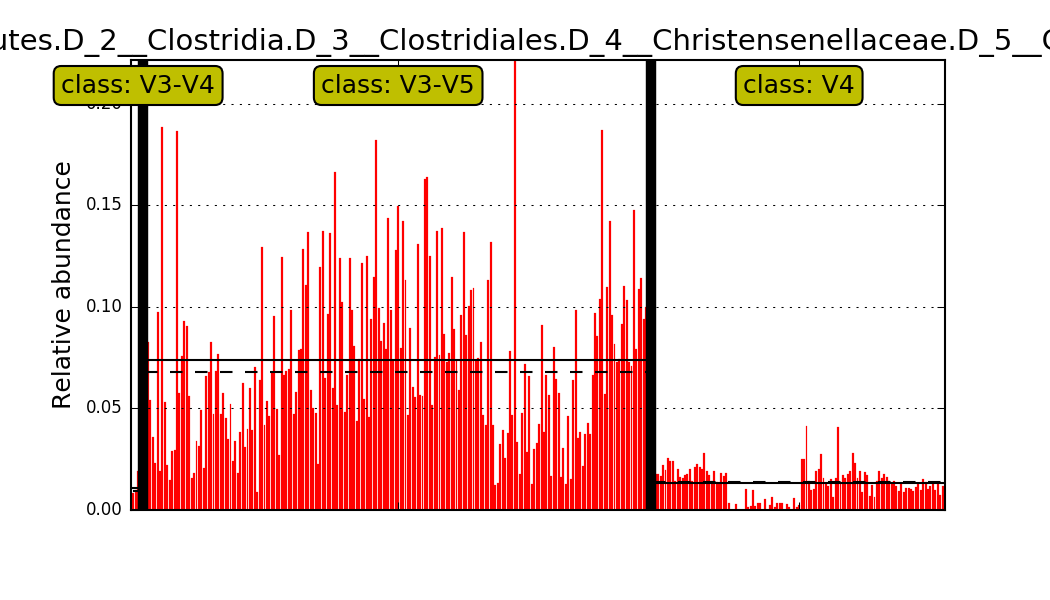


*Christensenellaceae* R7 group

*Eubacterium nodatum* group

*Mogibacterium*


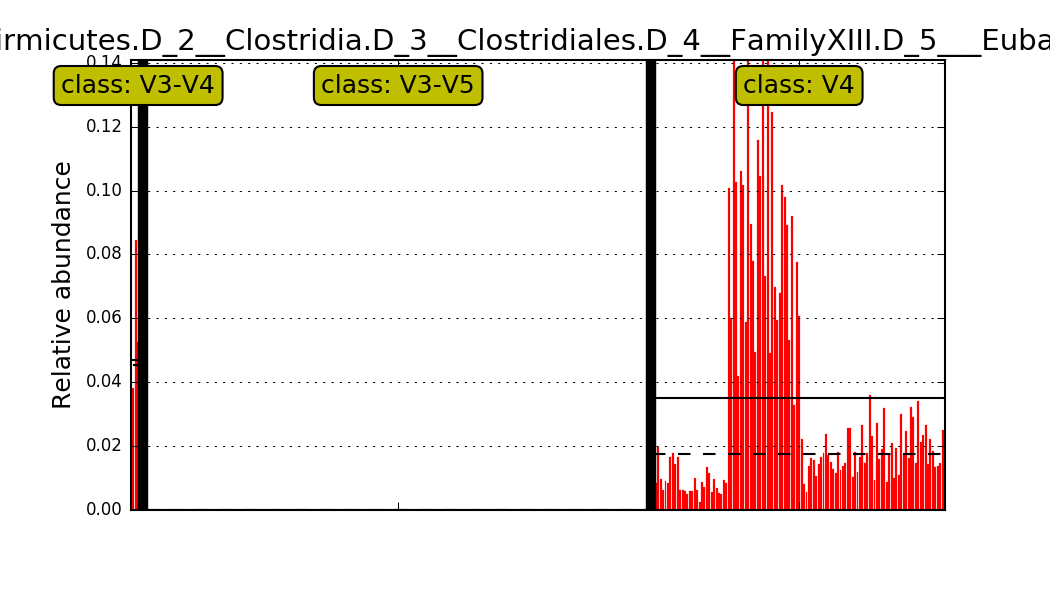

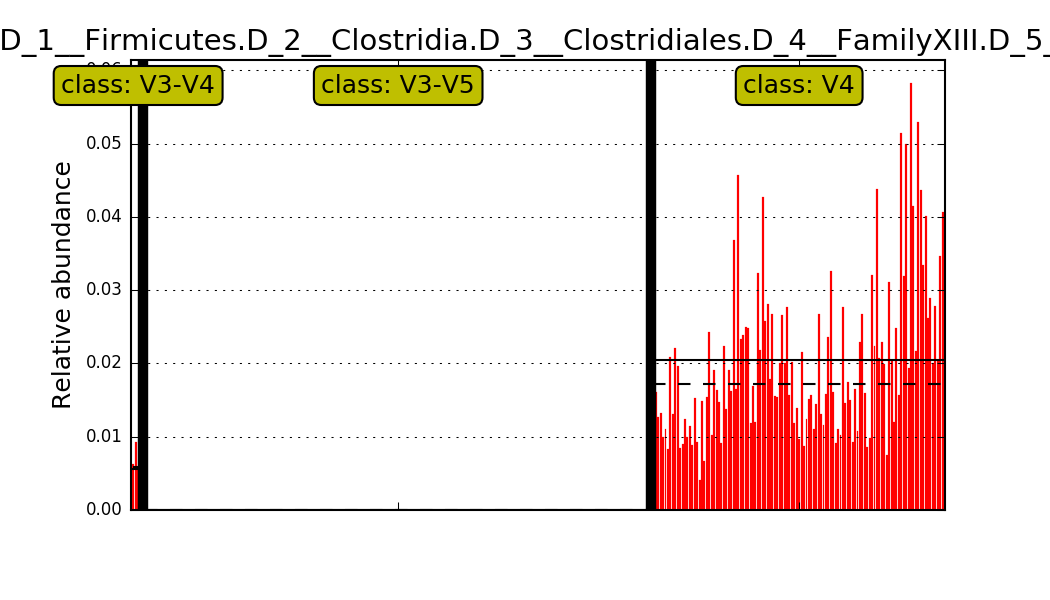

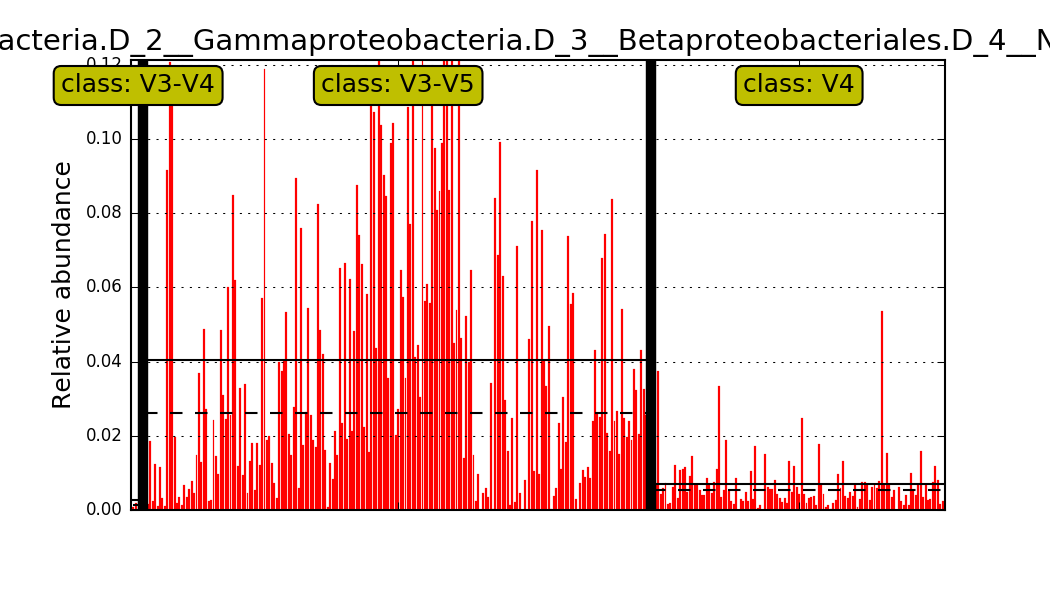


uncultured *Neisseriaceae*

*Butyrivibrio* 2


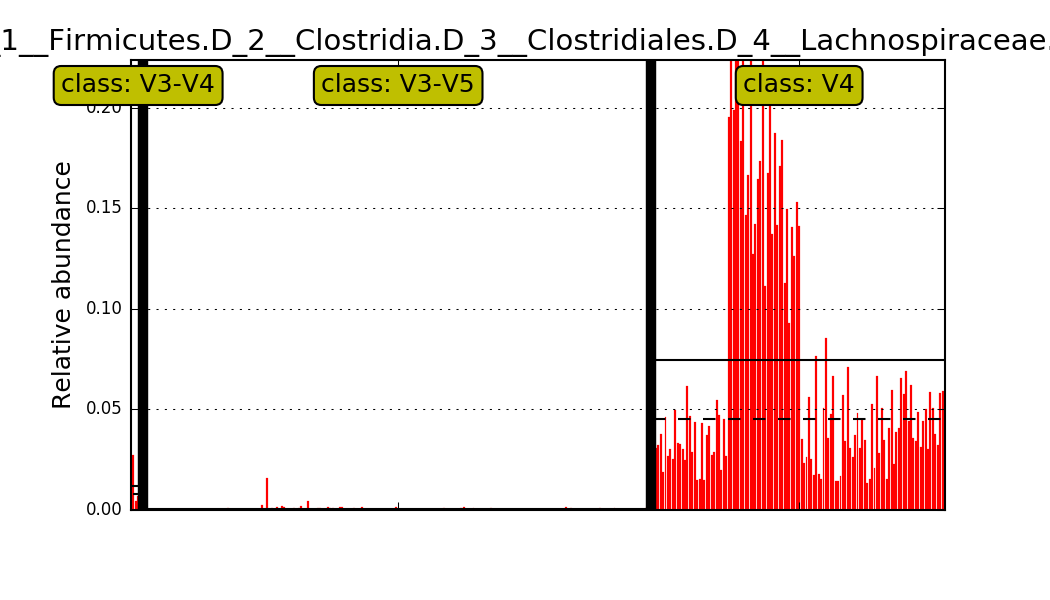

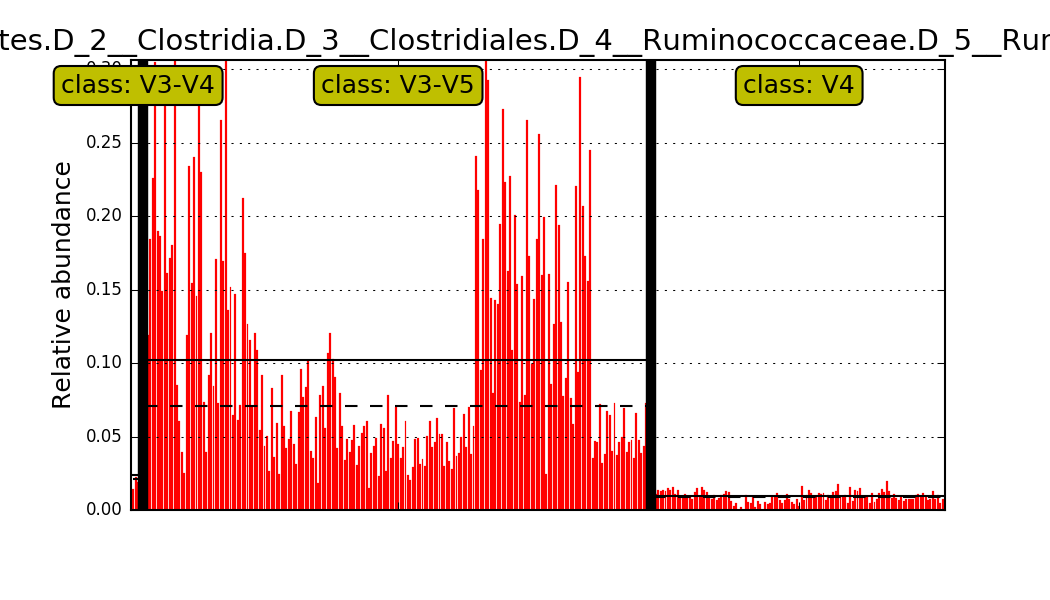


*Ruminococacceae* NK4A214 group

1. *utyrivibrio* 2


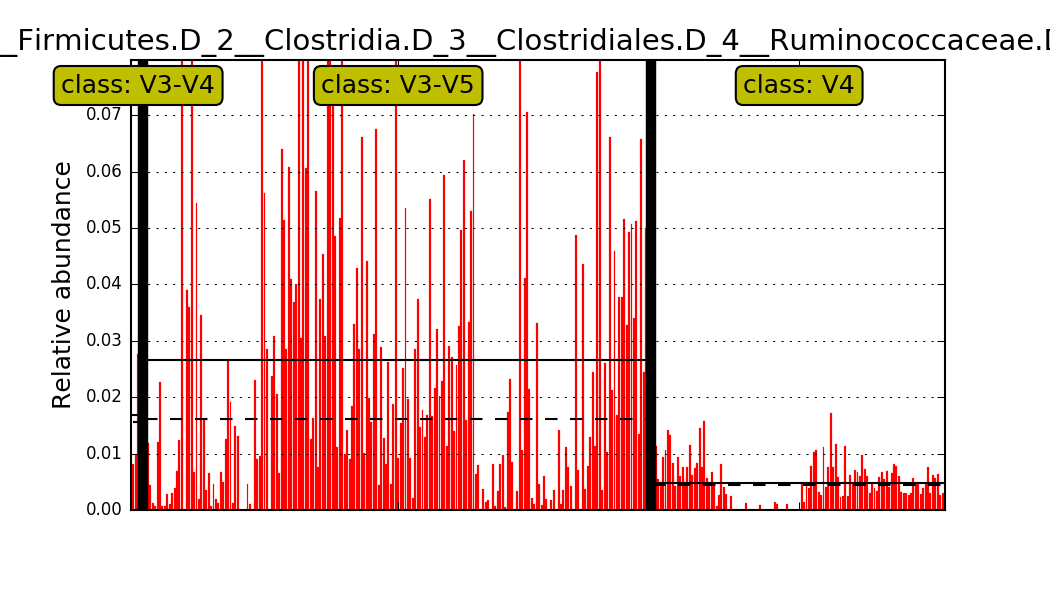


*Ruminococcus* 1


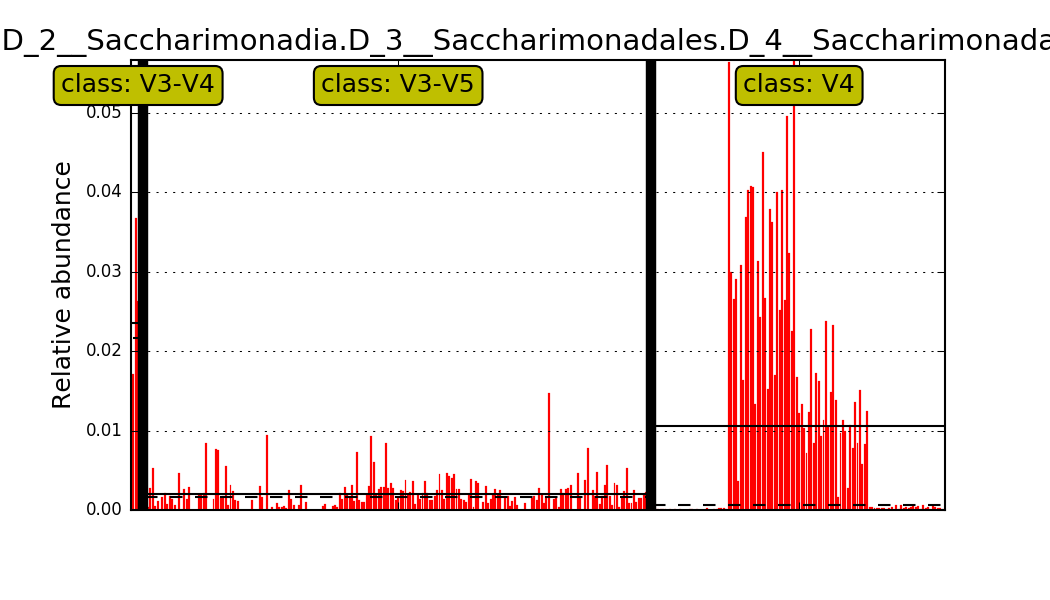


Candidatus *Saccharimonas*


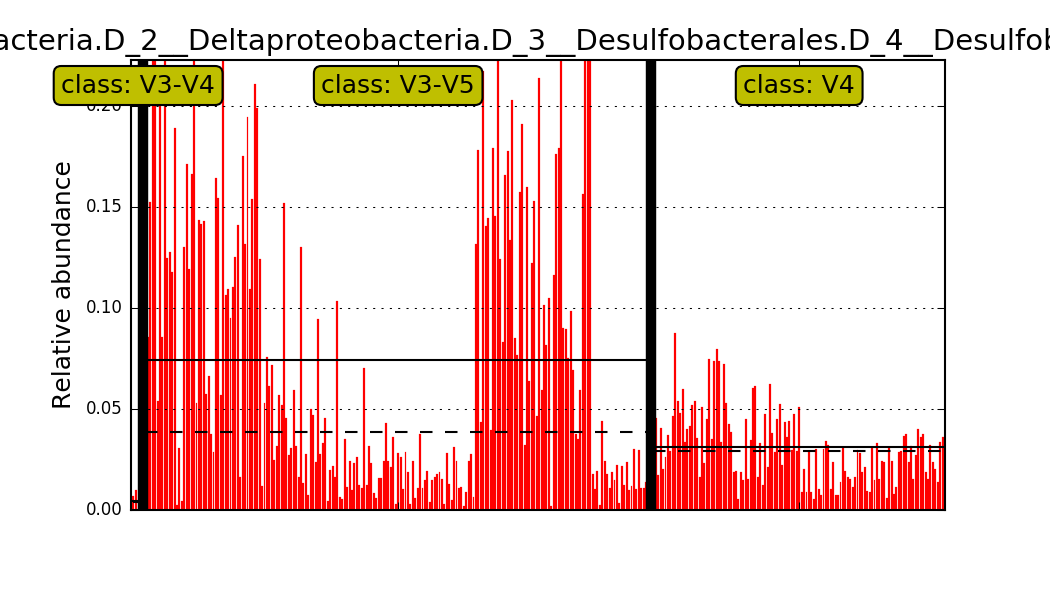


*Desulfobulbus*


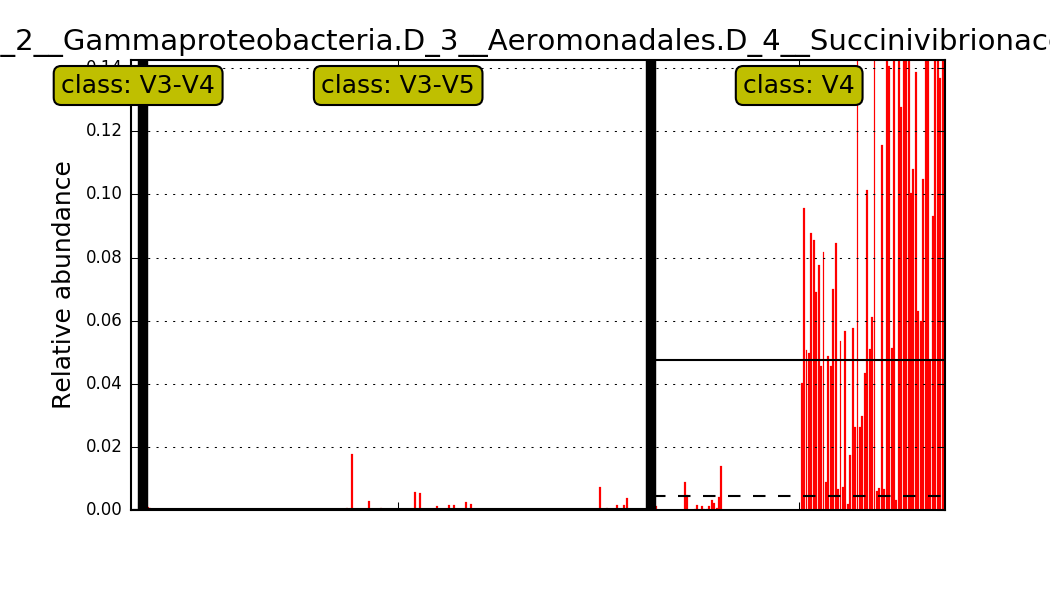


*Succinivibrionaceae* UCG-001


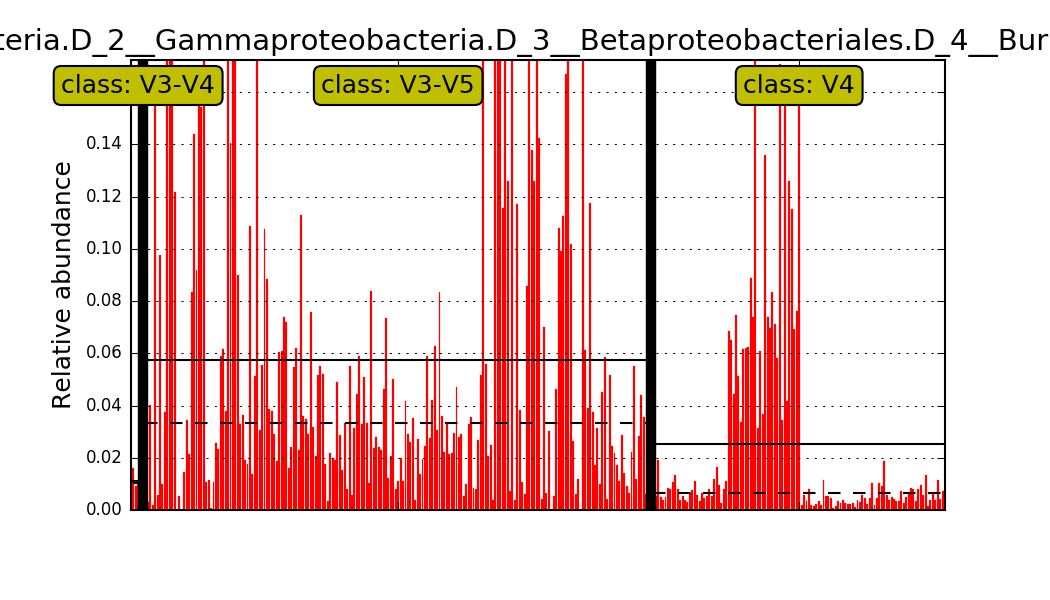


*Comamonas*

**Figure S2. Metabolic pathways identified by LEfSe as differentially abundant according to the biopsy location.**


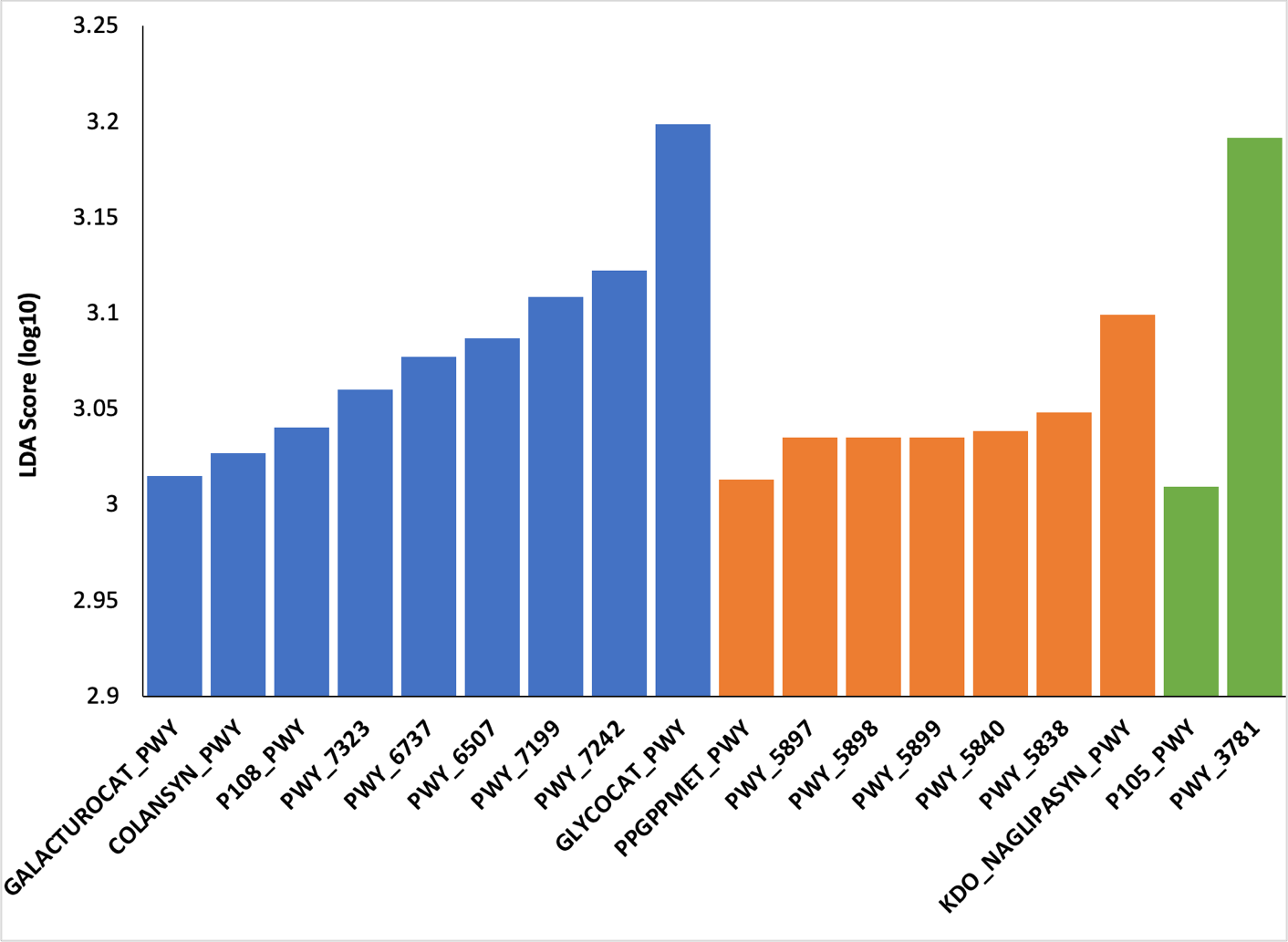

Supplement: Supplementary file 1 [file microorganisms-09-00342-s001.zip › Supplementary data_final.docx]
